# Targeting the proliferation of glioblastoma cells and enhancement of doxorubicin and temozolomide cytotoxicity through inhibition of PFKFB4 and HMOX1 genes with siRNAs

**DOI:** 10.1038/s41598-025-97192-z

**Published:** 2025-07-30

**Authors:** Hamzeh J. Al-Ameer, Malek Zihlif, Ahmed Maslat, Wajdy J. Al-Awaida, Amani Marwan Ayyash, Amer Imraish, Nidal Al-Qinna, Tareq Al-Omari, Talal Al-Qaisi, Walid Al-Zyoud, Bayan T. Alzubi, Ali M. Atoom, Isam A. Fattash, Shubhankar Ambike, Khang Wen Goh, Yulia Sh. Gushchina

**Affiliations:** 1 Department of Biotechnology, Faculty of Allied Medical Sciences, Al-Ahliyya Amman University, Amman, 19328 Jordan; 2 Department of Biological Sciences, Faculty of Science, Yarmouk University, Irbid, 21163 Jordan; 3 Department of Pharmacology, Faculty of Medicine, The University of Jordan, Amman, 11942 Jordan; 4 Department of Biology and Biotechnology, Faculty of Science, American University of Madaba, Madaba, 17110 Jordan; 5 Department of Pharmacy, Faculty of Health Sciences, American University of Madaba, Madaba, 17110, Jordan; 6 Department of Biological Sciences, School of Science, The University of Jordan, Amman, 11942 Jordan; 7 Pharmaceutical Center (UPPC), Faculty of Pharmacy and Medical Sciences, University of Petra, Amman, 11196 Jordan; 8 Department of Biomedical Sciences, College of Health Sciences, Abu Dhabi University, P.O. Box 59911, Abu Dhabi, UAE; 9 Department of Medical Laboratory Sciences, Faculty of Allied Medical Sciences, Al-Ahliyya Amman University, Amman, 19328 Jordan; 10 Department of Biomedical Engineering, School of Applied Medical Sciences, German Jordanian University, Amman, 11180 Jordan; 11 Institute of Virology, School of Medicine, Technische Universität München / Helmholtz Zentrum München, Trogerstr. 30, 81675 Munich, Germany; 12 Faculty of Data Science and Information Technology, INTI International University, Nilai, 71800 Malaysia; 13 Department of General and Clinical Pharmacology, Medical Institute, Peoples’ Friendship University of Russia (RUDN University), Moscow, 117198 Russia; 14 Duke-NUS Medical School, Programme in Emerging Infectious Diseases, 8 College Road, Singapore, 169857 Singapore

**Keywords:** Glioblastoma, siRNA, PFKFB4, HMOX1, Doxorubicin, Temozolomide, Human health, Cancer, Computational biology and bioinformatics, Drug discovery, Molecular biology, Neuroscience, Oncology

## Abstract

Glioblastoma multiforme continues to be one of the most aggressive brain cancers, posing a serious health challenge, as it offers a median survival of only 15–23 months and a 5-year survival rate of less than 6%. Current treatments often prove inadequate, underscoring the urgency for new therapeutic strategies. This study investigated the potential of silencing the PFKFB4 and HMOX1 genes in U87-MG glioblastoma cells using small interfering RNAs (siRNAs), both alone and alongside the chemotherapeutic agents temozolomide (TMZ) and doxorubicin (DOX). Through MTT assays, qPCR, and wound healing techniques, we assessed cell viability, gene expression, and cell migration. Notably, siPFKFB4 enhanced DOX’s cytotoxic effect, reducing its IC_50_ by six-fold, while having a milder impact with TMZ. When both siRNAs were combined with DOX, the IC_50_ decreased by two-fold without harming normal cells. Although siHMOX1 reduced cell migration, it only modestly affected cell proliferation with either DOX or TMZ. The gene expression analysis demonstrated that the siPFKFB4/DOX treatment led to an upregulation of pro-apoptotic genes such as DPYSL4, while simultaneously downregulating anti-apoptotic genes, including BCL-2 and PARP2. In contrast, the siHMOX1 combination influenced the expression of 14 genes, significantly enhancing the levels of CYLD, FAS, and CASP3, which are key promoters of apoptosis. In migration assays, siPFKFB4/DOX and siHMOX1/DOX reduced cell migration by 65 and 75%, respectively. These findings suggest that siPFKFB4 combined with DOX offers a promising pathway for GBM therapy, advocating further exploration into effective central nervous system drug delivery methods.

**Supplementary Information:**

The online version contains supplementary material available at 10.1038/s41598-025-97192-z.

## Introduction

Glioblastoma Multiforme (GBM), classified as a grade IV astrocytoma, is recognized as the most common, aggressive, and invasive type of brain tumor. It originates in the star-shaped glial cells that provide support to nerve cells in the brain and constitutes more than 15% of all primary brain tumors. Glioblastoma can manifest in two distinct forms. Primary glioblastoma typically occurs in individuals of all ages, though the median age is 60 years, exhibits a rapid progression, often developing within a few months^1^. In contrast, secondary glioblastomas could emerge from low-grade gliomas, primarily affecting younger individuals, even under the age of 45. GBM is distinguished by its capacity to penetrate normal brain tissue, making surgical removal and treatment extremely challenging^2^. Despite advancements in contemporary treatments for GBM, it continues to be a deadly disease with a notably poor prognosis. Patients diagnosed with GBM have a reported median survival of around 15–23 months, and the 5-year survival rate for malignant brain tumors stands at approximately 7%, reflecting one of the lowest long-term survival rates among cancers^3^. More than 90% of individuals undergoing treatments could have a recurrence within 2 years with a life expectancy of 5–7 months^4^. Such a low survival rate has been associated with a rapid tumorigenesis of cancerous cells along with invasion of nearby brain tissue^5^. Fractionated radiotherapy and temozolomide (TMZ) are the gold standard of GBM treatment. However, because of development of chemoresistance, recurrent GBM poses a significant challenge to establish alternative treatment strategies^4^. As a result, novel and efficient alternative therapeutic strategies to treat GBM are of urgent need^5^.

In positing novel therapeutics for GBM, targeted therapies have emerged as a promising avenue, these therapies are meticulously crafted to focus on the underlying genetic and molecular pathways that propel tumor growth^6^. With cell-specific interference of such pathways, targeted therapies could offer a potential breakthrough in addressing the complexities of GBM treatment^7^. A notable example is the targeting of the PI3K/AKT/mTOR pathway using the small molecule inhibitor BEZ235, which has proven effective in inhibiting the growth of GBM cells in both in vitro and in vivo studies^8^. The excessive metabolic activation of the PI3K/AKT/mTOR pathway has been linked to the suppression of apoptosis and the increased proliferation of cancer cells, as observed in various malignancies, including gliomas. Consequently, identifying and targeting this pathway presents a promising therapeutic strategy for treating GBM^8^.

The selection of Heme oxygenase-1 (HMOX1) and the enzyme 6-phosphofructo-2-kinase/fructose-2,6-biphosphatase 4 (PFKFB4) as therapeutic targets is grounded in their pivotal roles in promoting tumor progression and treatment resistance. PFKFB4, in particular, plays a crucial role in glucose metabolism within cancer cells by facilitating the reversible conversion of fructose-2,6-bisphosphate to fructose-6-phosphate, a key step in the glycolysis pathway. Its involvement in regulating the cell cycle and apoptosis makes PFKFB4 a promising target for therapy^9^. Previous studies have shown that inhibiting PFKFB4 disrupts glucose metabolism in cancer cells, offering a potential treatment strategy for glioblastoma^10^.

HMOX1, an enzyme crucial for heme breakdown, is implicated in the progression of several cancers, including glioblastoma. HMOX1 supports rapid tumor growth, cancer aggressiveness, angiogenesis, and metastasis^11^. Byproducts of HMOX1 activity also modulate the immune response; making it a valuable therapeutic target. Inhibition of HMOX1 has been demonstrated to increase the efficacy of chemotherapeutic agents, such as doxorubicin in breast cancer and temozolomide (TMZ) in melanoma, enhancing their therapeutic impact^12^. This is consistent with studies showing that PFKFB4 and HMOX1 are overexpressed in various cancer types and are linked to poorer prognosis and reduced survival rates in brain cancer patients^13^.

Considering the critical roles of these two genes, this study investigated the potential of targeting PFKFB4 and HMOX1 as promising therapeutic strategies for the treatment of GBM. We primarily investigated the additive anti-proliferative and anti-migratory effects of HMOX1 and PFKFB4 knockdown alone or in combination with temozolomide (TMZ) and doxorubicin in U87-MG Glioblastoma Cells. By studying U87-MG glioblastoma cells, we aimed to identify the genes involved in cell death following siRNA treatment and chemotherapy. Our research shows how silencing PFKFB4 and HMOX1 can help in GBM therapy and suggests a new targeted treatment option.

## Methodology approaches and the experimental materials

### Procedures for culturing and expanding cell lines

The U-87 MG glioblastoma and HDFa fibroblast cell lines were obtained from ATCC (Manassas, VA, USA) and cultured following standard protocols. U-87 MG cells were maintained in high-glucose DMEM, and HDFa cells in IMDM, both supplemented with 10% FBS, penicillin, streptomycin, and amphotericin B. Cells were incubated at 37 °C with 5% CO₂ and sub-cultured upon reaching 80% confluency using 0.25% trypsin/EDTA. Culture conditions and media preparation adhered to established methods^14^.

### Cell viability and counting

Cell viability was assessed using the trypan blue exclusion method. A 1:1 mixture of cells and trypan blue was prepared, loaded onto a hemocytometer, and analyzed under an inverted microscope (Leica Microsystems) at 100× magnification to distinguish viable cells from those with compromised membranes^14^.

### siRNA design and transfection

Custom-designed 27-mer siRNAs targeting PFKFB4 (**SR303465**) and HMOX1 (**SR320506)** (OriGene Technologies) were prepared according to the manufacturer’s instructions. Transfections were performed using Lipofectamine RNAiMAX (Invitrogen) in serum-free media, following the recommended protocol. A scrambled siRNA (OriGene) served as a control to verify transfection efficiency and minimize off-target effects. Six hours post-transfection, 10% FBS was added to the culture media to promote optimal cell growth^15^.

The rationale for using a 100 nM siRNA concentration in most experiments is to ensure a clear and consistent impact on gene expression, as the timing of cell harvesting varied across experiments. For instance, IC_50_ values were determined after 72 h, while PCR array experiments were conducted after 24 h. Using 100 nM also ensured a homogenous cell population progressing toward apoptosis, thereby providing clear insights into cellular responses to siRNA treatment.

To ensure the specificity of siRNA knockdown, scrambled siRNA controls were included in all experiments. Off-target effects were evaluated by monitoring the expression levels of housekeeping genes, which showed no significant changes throughout the study. Biological replicates were performed across multiple time points to confirm reproducibility. These measures provided robust validation of the experimental design and knockdown efficacy.

### Cytotoxicity evaluation of siPFKFB4 and siHMOX1

U-87 MG and HDFa cell lines were seeded into 96-well plates at densities of 5 × 10³ and 7 × 10³ cells per well, respectively, and cultured under standard conditions to allow adherence for 24 h. Transfections with siPFKFB4, siHMOX1, and a scrambled control were performed in serum-free media using Lipofectamine RNAiMAX (Invitrogen) across a concentration range of 0.8–400 nM. After 6 h, the media were replaced with complete media to maintain cell health^16^.

Cytotoxic effects were assessed 72 h post-transfection using the MTT assay (Promega), following the manufacturer’s instructions. Absorbance was measured at 570 nm using a Synergy™ HTX Microplate Reader (BioTek). IC_50_ values were calculated using GraphPad Prism Software 8.0.1 with a variable slope model^16^.

### Evaluating in vitro cytotoxic effects of doxorubicin or temozolomide in combination with siPFKFB4 or siHMOX1

The synergistic effects of doxorubicin (DOX; Ebewe Pharma, Australia) and temozolomide (TMZ; Sigma Aldrich) with siPFKFB4 or siHMOX1 were evaluated in U-87 MG and HDFa cells using the MTT assay. Cells were transfected with siRNAs at a fixed concentration of 100 nM, followed by treatment with serial dilutions of DOX (0.025–12.5 µM) or TMZ (3.9–2000 µM)^16,17^.

After transfection and drug exposure, cells were incubated for 72 h under normoxic conditions (5% CO₂, 37 °C) in a humidified incubator. All experiments were performed in triplicate^16^.

### In vitro cytotoxicity assay of doxorubicin and temozolomide

The cytotoxicity of doxorubicin (DOX; Ebewe Pharma) and temozolomide (TMZ; Sigma Aldrich) was evaluated in U-87 MG and HDFa cells using the MTT assay. DOX was prepared as a 2 mg/ml stock solution in sterile water and stored at 4 °C, while TMZ was prepared as a 100 mM stock solution in DMSO and stored at − 20 °C, with the final DMSO concentration kept below 0.1% to minimize toxicity^14^.

Cells were seeded in 96-well plates at densities of 5 × 10³ (U-87 MG) and 7 × 10³ (HDFa) cells per well in 100 µL of culture medium and incubated under normoxic conditions (5% CO₂, 37 °C) for 24 h to allow adherence. Treatments with varying concentrations of DOX (0.05–25 µM) or TMZ (3.9–2000 µM) were applied for 72 h, and all experiments were performed in triplicate. Cell viability was assessed using the MTT assay as per the standard protocol^14^.

### Control groups

Control groups were included to ensure result accuracy and reliability. Positive controls were treated with known concentrations of doxorubicin (DOX) and temozolomide (TMZ) to confirm expected cytotoxic effects. Negative controls included untreated cells and cells treated with scrambled siRNA to identify non-specific effects, while untreated controls provided baseline measurements for cell viability and gene expression. These controls were critical for interpreting the experimental results and verifying the specific effects of siRNA and drug treatments.

### Determination of IC_50_

The IC_50_ values for DOX (0.025–12.5 µM) and TMZ (3.9–2000 µM) were determined using GraphPad Prism Software (v8.0.1). TMZ was freshly prepared in DMSO (ChemCruz), with a final DMSO concentration maintained at ≤ 1%. Each treatment condition was tested in triplicate, and the IC_50_ values were calculated using a variable slope model^14^.

### RNA extraction and cDNA synthesis

Total RNA was extracted using the RNeasy Plus Mini kit (Qiagen) per the manufacturer’s instructions and stored at − 80 °C. For cDNA synthesis, 1 µg of RNA was reverse transcribed using the RT2 First-Strand Synthesis Kit (Qiagen), following the manufacturer’s protocol^14^.

### Assessing gene expression levels using quantitative RT-PCR

Gene expression levels of PFKFB4 and HMOX1 were analyzed in U87-MG and HDFa cell lines using RT-qPCR, with GAPDH as the housekeeping gene. The reactions were conducted on the C1000 Touch™ Thermal Cycler/CFX96™ Real-Time System (Bio-Rad) using TB Green Premix Ex Taq™ II reagent (Takara). The 2^−ΔΔCt method was employed for relative quantification of mRNA expression levels. Primer sequences and additional details are available in Table 1^18^.

**Table 1:** List of the primer sequences and annealing temperature.

Gene	Forward primer	Reverse primer	Ta	Ref.
PFKFB4	GGGTGCCTCTTGGCCTTAAA	GCCCACACGGCATACTTTTC	62 °C	^18^
HMOX1	AAGACTGCGTTCCTGCTCAAC	AAAGCCCTACAGCAACTGTCG	62 °C	^19^
GAPDH	CTCTGATTTGGTCGTATTGGG	TGGAAGATGGTGATGGGATT	62 °C	^18^

Ta: Annealing Temperature

Ref: Reference

#### Evaluating U87-MG cell migration through an in vitro scratch wound healing assay

The migratory ability of U87-MG cells was assessed using an in vitro scratch wound healing assay. Approximately 1 × 10⁵ cells were seeded into pre-treated 6-well plates containing high-glucose DMEM and cultured to 80–90% confluence. A scratch was created using a P200 pipette tip, and wound closure was monitored at 0, 24, 48, and 72 h using an inverted microscope with a camera (Leica Microsystems). Wound closure percentages were analyzed with Fiji software, calculated as:

% of wound closure = [(A_t=0 h_ – A_t=∆h_)/A_t=0 h_] X 100%,

where A_t=0 h_ represents the wound area measured immediately after scratching, and A_t=∆h_ denotes the wound area at 24, 48, and 72 h post-scratch^21^.

### Gene expression profiling and real-time PCR array

Pathway-specific gene expression profiling was performed using the RT2 Profiler PCR Array (Qiagen, PAHS-212ZA), which targets 84 genes related to apoptosis, autophagy, and necrosis. A detailed list of the genes is provided in the supplementary material (Table S1). To prevent RNase contamination, all surfaces and equipment were treated with RNaseZap^®^ (ThermoFisher).

For each 96-well array plate, 1 µg of cDNA, prepared from total RNA, was mixed with RT2 SYBR^®^ Green Master Mix (Qiagen) and loaded into the wells. Real-time PCR was conducted on the C1000 Touch™ Thermal Cycler/CFX96™ System (Bio-Rad) using standard amplification conditions: 95 °C for 10 min, followed by 40 cycles of 95 °C for 15 s and 60 °C for 1 min^14^.

### Data analysis of gene expression profiling

Gene expression analysis was performed using the 2^−ΔΔCt method with data from the PCR array. Fold changes in gene expression were calculated through the automated web portal provided by SA Biosciences (Qiagen) at www.SABiosciences.com/pcrarraydataanalysis.php. Normalization was carried out using reference genes RPLP0, ACTB, B2M, and GAPDH to ensure consistency across all plates.

### Bioinformatics analysis

RNA sequencing data from the TCGA GBM cohort were analyzed using TIMER and GEPIA2 databases to investigate PFKFB4 and HMOX1 expression in malignant and normal tissues. Kaplan-Meier survival analysis assessed overall survival (OS), disease-free survival (DSS), and progression-free interval (PFI) based on gene expression levels, with statistical significance determined by the log-rank test (*p* < 0.05). Hazard ratios (HRs) and 95% confidence intervals (CIs) were calculated to evaluate these relationships.

Co-expression patterns of PFKFB4 and HMOX1 were examined using Spearman’s correlation (*p* ≤ 0.05, FDR ≤ 0.05). Gene Set Enrichment Analysis (GSEA) identified functional associations through Gene Ontology (GO) terms and Kyoto Encyclopedia of Genes and Genomes (KEGG) pathways^22^. A CDKN3-related protein-protein interaction (PPI) network was constructed using the STRING database^23^.

### Statistical methods and data analysis

All experiments were performed in triplicate unless stated otherwise. Results are expressed as mean ± SEM, with *p* < 0.05 considered statistically significant. Statistical analyses were conducted using GraphPad Prism Software (v8.0.1).

## Results

### Assessment of cytotoxic activity in U87-MG cells through in vitro assays

**Fig. 1:**
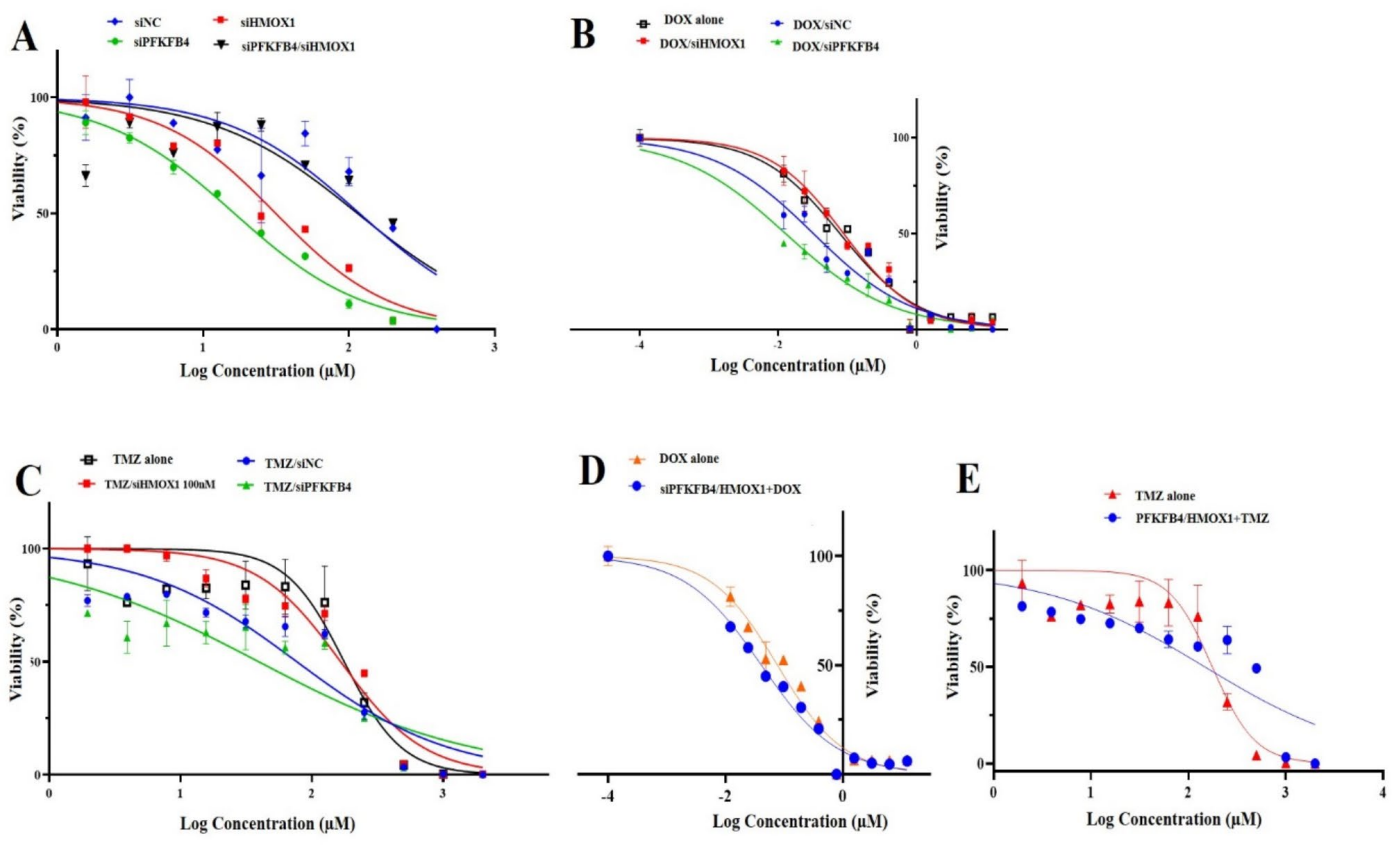
In vitro cytotoxicity assay analysis of U87-MG cells for 72 h. (**A**) The effect of siRNAs only against U87-MG cell viability. (**B**) The effect of serial does of doxorubicin (DOX) alone or in combination with 100 nM of siRNAs. (**C**) The effect of serial does of temozolomide (TMZ) alone or in combination with 100 nM of siRNAs. (**D**) The effect of serial doses of doxorubicin (DOX) alone or in combination with both siRNAs (siPFKFB4 and siHMOX1). (**E**) The effect of serial doses of temozolomide (TMZ) alone or in combination with both siRNAs (siPFKFB4 and siHMOX1).

Dose-dependent viability assay for each siRNA (siPFKFB4, siHMOX1, and Scrambled), as well as both siPFKFB4/siHMOX1 was performed with serial doses of siRNAs ranging from 0.8 to 400 nM for 72 h. The viability values were compared with scrambled siRNA as a negative control and untreated control cells as expressed with normalized viability of the used concentrations. We found that IC_50_s were 16.33 ± 1.50 nM for siPFKFB4, 31.40 ± 0.01 nM for siHMOX1, siPFKFB4/siHMOX1 108.8 ± 3.1 nM, and 118.2 ± 0.50 nM for Scrambled (Fig. 1A). Although the IC_50_ values for siPFKFB4 (16.33 nM) and siHMOX1 (31.40 nM) were determined, a concentration of 100 nM siRNA was chosen for subsequent experiments. This concentration was selected to ensure robust and consistent knockdown efficiency across all experimental setups, particularly in co-treatment conditions with chemotherapeutic agents. The choice also aligns with established protocols in similar studies, balancing efficacy and potential off-target effects.

The In vitro MTT assay demonstrated that the chemo-sensitivity of DOX against the glioblastoma cell line was increased, and the IC_50_ was decreased. The combination of serial doses of DOX with 100 nM of siPFKFB4 decreased the IC_50_ value by 6 times over DOX alone from 0.078 ± 0.014 to 0.013 ± 0.0001 µM. Moreover,. The combination of serial doses of DOX with 100 nM siHMOX1combination treatment resulted in an IC_50_ value of 0.091 ± 0.0001 µM, which had a less significant effect on glioblastoma cells. On the other hand, DOX-siNC exhibited a double reduction over the IC_50_ value of DOX alone from 0.078 ± 0.014 to 0.033 ± 0.0014 µM (Fig. 1B).

The In vitro MTT assay showed that the chemo-sensitivity of TMZ against the glioblastoma cell line was increased, and the IC_50_ was decreased. The combination of serial doses of TMZ with 100 nM of siPFKFB4 decreased the IC_50_value by 4 times over TMZ alone from 175.90 ± 3.71 to 39.74 ± 0.49 µM. Moreover, The combination of serial doses of TMZ with 100 nM of siHMOX1 combination treatment had no significant effect on glioblastoma cells. On the other hand, DOX-siNC exhibited a double reduction over the IC_50_ value of TMZ alone from 175.90 ± 3.71 to 77.14 ± 0.48 µM (Fig. 1C).

The In vitro MTT assay showed that the chemo-sensitivity of DOX against the glioblastoma cell line was significantly increased, and the IC_50_ was decreased. The combination of siPFKFB4/siHMOX1 + DOX resulted in a two-fold reduction in IC_50_ on glioblastoma cells compared to the DOX alone, from 0.078 ± 0.014 to 0.042 ± 0.001 µM (Fig. 1D).

The In vitro MTT assay showed that the chemo-sensitivity of TMZ against the glioblastoma cell line was increased, and the IC_50_ was decreased. The combination of siPFKFB4/HMOX1 + TMZ resulted in an IC_50_ value of 147.9 µM, showing a moderate reduction in glioblastoma cells compared to the TMZ alone IC_50_ value of 175.90 ± 3.71 µM, as shown in (Fig. 1E).

### Toxicity assay on normal fibroblast cells (HDFa)

**Fig. 2:**
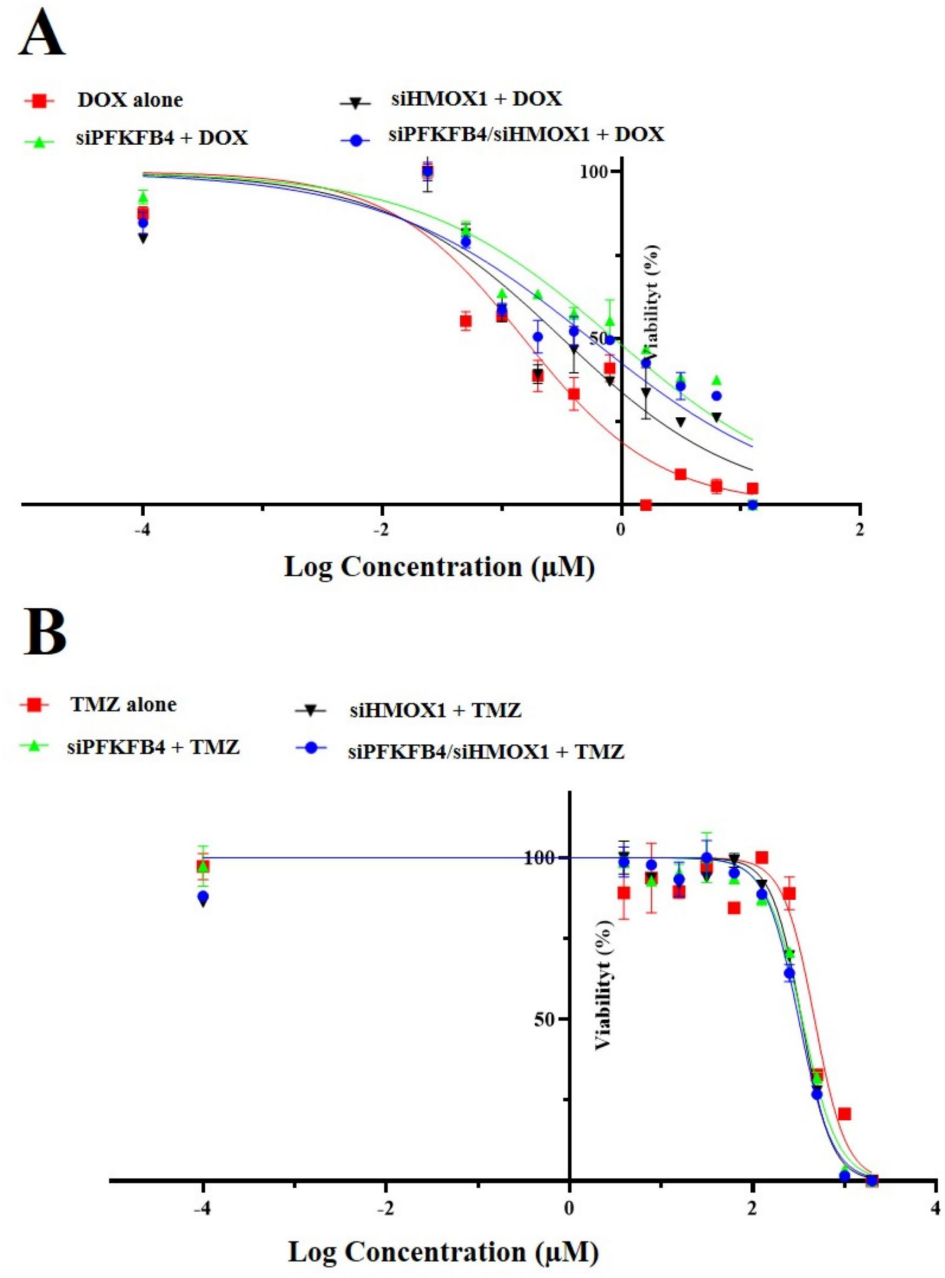
In vitro cytotoxicity assay analysis on HDFa cells for 72 h. (**A**) The effect of serial does doxorubicin (DOX) alone, or with 100 nM of each siRNAs either separately or both siRNAs in combination with DOX. (**B**) The effect of serial does temozolomide (TMZ) alone, or with 100 nM of each siRNAs either separately or both siRNAs in combination with TMZ.

The In vitro MTT assay showed that the chemo-sensitivity of DOX alone (0.16 ± 0.01 µM) against the HDFa cell line was low, and the IC_50_ was high. On the contrary, the combination of siPFKFB4 + DOX (1.01 ± 0.20 µM), siHMOX1 + DOX (0.33 ± 0.02 µM), and siPFKFB4/siHMOX1 + DOX (0.61 ± 0.09 µM) respectively has a less toxic effect than DOX alone on the HDFa cell line, as shown in (Fig. 2A**)**.

The In vitro MTT assay showed that the chemo-sensitivity of TMZ alone (470.20 ± 7.68 µM) against the HDFa cell line was low, and the IC_50_ was high. As opposed to TMZ alone, siPFKFB4 + TMZ (345.93 ± 21.88 M), siHMOX1 + TMZ (333.43 ± 8.73 M), and siPFKFB4/siHMOX1 + TMZ (313.31 ± 4.52 M) have similar effects on HDFa cells, as shown in (Fig. 2B**)**.

### Quantitative evaluation of gene expression levels via RT-qPCR

**Fig. 3:**
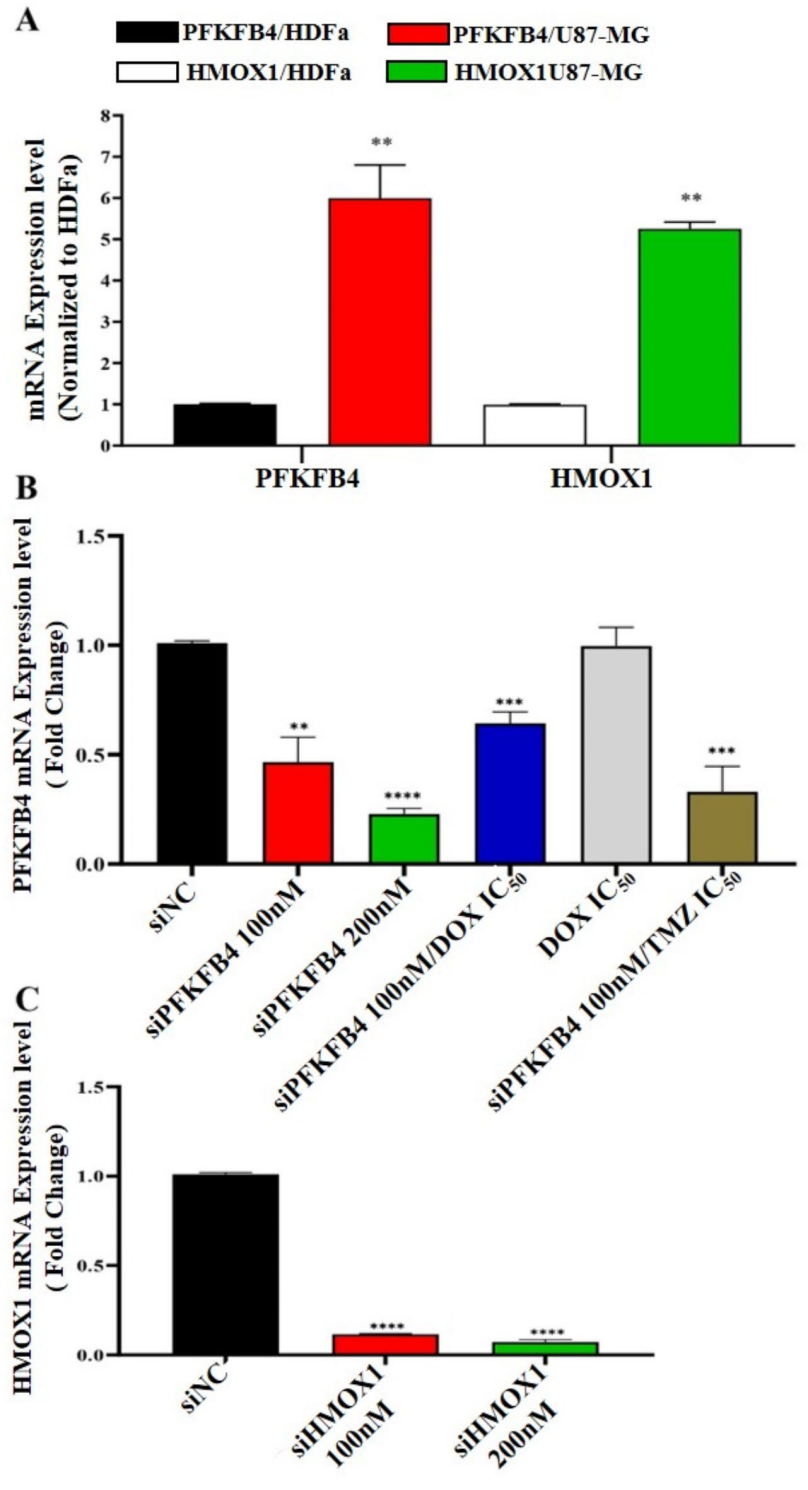
mRNA knockdown efficacy of siRNA treatments, as measured by qPCR. This figure demonstrates the silencing efficacy of siRNA treatments, with strong mRNA-to-protein correlation supported by prior studies. While protein-level validation was not performed due to resource constraints, qPCR results provide reliable evidence of knockdown efficacy.

The mRNA expression levels of the PFKFB4 and HMOX1 genes were assessed in both U-87 MG glioblastoma and HDFa normal cell lines using RT-qPCR. The analysis revealed a significant upregulation of PFKFB4 and HMOX1 expression in the glioblastoma cells compared to the normal cells. For comparative purposes, the expression levels in the HDFa cells served as the control reference for this experiment, as depicted in Fig. 3A. To confirm the knockdown efficiency, RT-qPCR was employed to measure the mRNA expression levels of the PFKFB4 gene in U-87 MG cells following treatment with siPFKFB4 at concentrations of 100 nM and 200 nM.

Therefore, the mRNA level of the *PFKFB4* gene has been down-regulated by 2.17-folds and 4.43-folds, respectively. In addition, 100 nM of PFKFB4 siRNA with DOX IC_50_ and 100 nM of PFKFB4 siRNA with TMZ IC_50_ has been down-regulated the mRNA level of the *PFKFB4* gene by 1.59-fold and 3.09-folds, respectively. Nevertheless, at DOX IC_50_ alone had no significant effect on the gene expression level of the *PFKFB4* gene. However, for a comparison between groups, the *scrambled* siRNA was considered a negative control. As shown in (Fig. 3B**)**.

RT-qPCR was used to determine the mRNA expression levels of *HMOX1* gene in the U-87 MG cells to ensure gene knockdown by using siHMOX1 at 100nM and 200nM concentrations. Therefore, the mRNA level of the *HMOX1* gene has been down-regulated by 8.59-folds and 13.99-folds, respectively. However, for a comparison between groups, the scrambled siRNA was considered a negative control. As shown in (Fig. 3C**)**. GAPDH was used as a reference gene, and the 2 − ΔΔCT method was employed to calculate the changes in gene expression levels. Figure 3 highlights the mRNA knockdown efficacy of siRNA treatments. Although protein-level validation was not performed, the observed mRNA changes are strongly correlated with protein expression levels for PFKFB4 and HMOX1, as supported by prior literature, ensuring the reliability of these findings.

(A) mRNA expression levels of **PFKFB4** and **HMOX1** genes in U87-MG cells compared to HDFa cells.
(B) Effect of treating U87-MG cells with 100 nM siPFKFB4, 200 nM siPFKFB4, siPFKFB4 combined with DOX (IC_50_), siPFKFB4 combined with TMZ (IC50), and scrambled siRNA (siNC) on the expression level of the **PFKFB4** gene.
(C) Effect of treating U87-MG cells with 100 nM siHMOX1, 200 nM siHMOX1, and scrambled siRNA (siNC) on the expression level of the **HMOX1** gene.

Data are presented as mean ± SEM. Statistical significance is indicated as *P* < 0.05 (*)*,  *P* < 0.01 (***)***,  *P* < 0.001 (), and *P* < 0.0001 (****). Abbreviations: siHMOX1, small interference HMOX1; siNC, small interference negative control.

### In vitro scratch wound healing assay

**Fig. 4:**
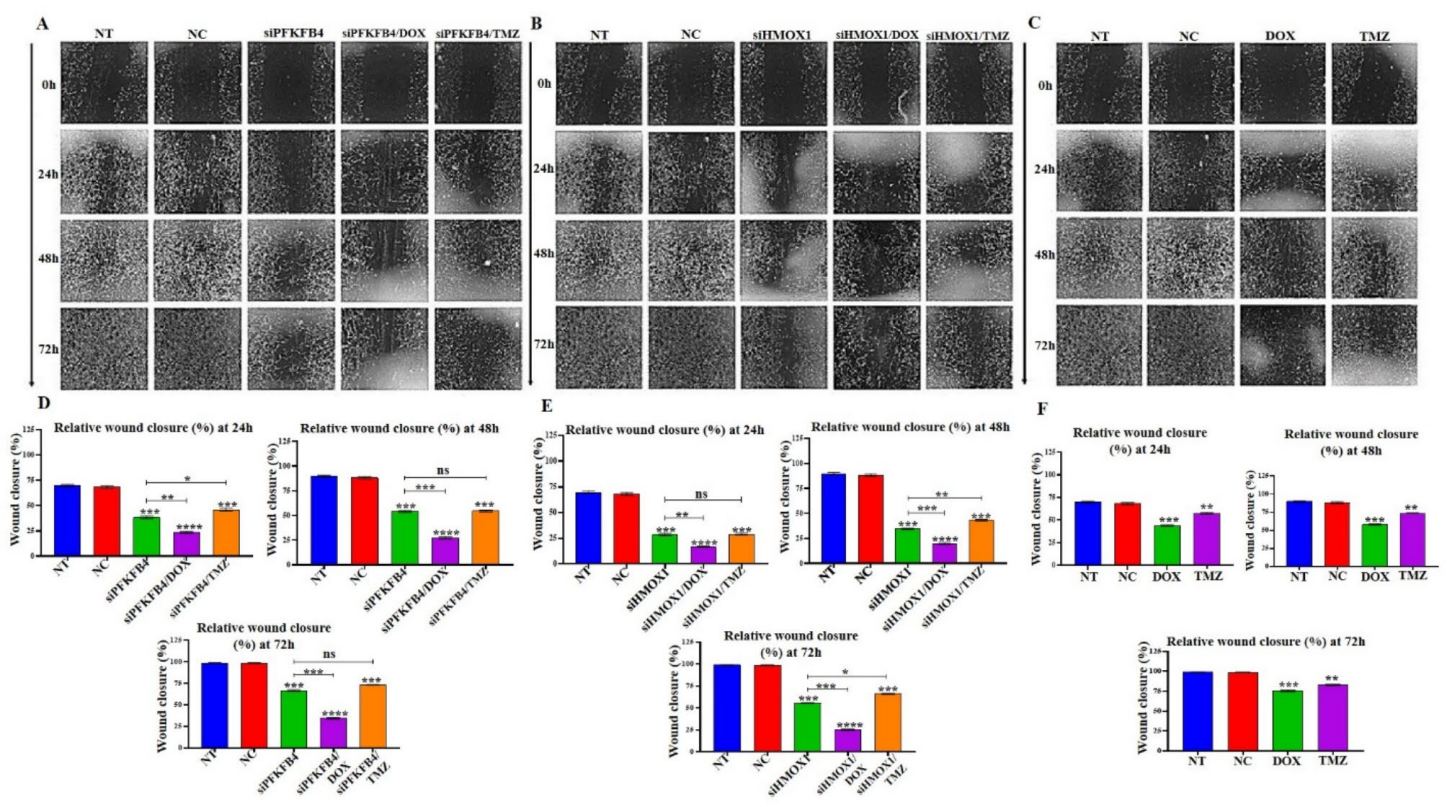
(**A**,** B**, and **C**) showcase images from a wound healing assay conducted at 0, 24, 48, and 72 hours following transfection with siPFKFB4, siHMOX1, and the chemotherapeutic agents DOX and TMZ in the U-87 MG cell line. The results indicate that silencing of PFKFB4 and HMOX1 mRNA through siPFKFB4 and siHMOX1 significantly hindered the migration of U-87 MG cells (magnification, X20). In panels (D, E, and F), bar charts quantify the extent of reduced migration in U-87 MG cells after silencing PFKFB4 and HMOX1 at the specified time points of 24, 48, and 72 hours post-transfection and treatment with DOX and TMZ. Statistical significance was determined using Student’s t-test, with data presented as mean ± SEM; significance levels are indicated as * *P* ≤ 0.05, ** *P* ≤ 0.01, *** *P* ≤ 0.001, and **** *P* ≤ 0.0001 in comparison to the negative control group. Here, ‘NT’ refers to nontransfected cells, ‘NC’ denotes cells transfected with negative control siRNA, and ‘siPFKFB4’ and ‘siHMOX1’ indicate cells treated with specific siRNAs targeting PFKFB4 and HMOX1. Abbreviations used include: siRNA, small interfering RNA; U-87 MG, glioblastoma cell line; PFKFB4, 6-Phosphofructo-2-Kinase/Fructose-2,6-Biphosphatase 4; HMOX1, heme oxygenase 1; DOX, doxorubicin; and TMZ, temozolomide.

The wound healing assay showed that the siPFKFB4 + DOX group showed a slower scratch healing speed on U87-MG cells compared with the NC group; the percentage of closure was 23.44 ± 1.16%, 27.44 ± 1.16%, and 34.59 ± 1.02% at 24 h, 48 h, and 72 h, respectively, as shown in Fig. 4A, D. Interestingly, the wound healing assay showed that the siHMOX1 + DOX group showed a more potent effect against U87-MG cell migration compared with the NC group; in which the percentage of closure was 16.67 ± 0.61%, 20.01 ± 0.87%, and 25.01 ± 0.87% at 24 h, 48 h, and 72 h, respectively, as shown in Fig. 4B, E. However, statistical analysis revealed no significant effect on GBM cell migration by chemotherapeutic agents (DOX and TMZ) alone. In which the percentage of closure for DOX was 43.96 ± 0.72%, 57.96 ± 0.95%, and 75.41 ± 1.31%, and for TMZ was 57.41 ± 0.95%, 73.74 ± 0.63%, and 82.96 ± 0.95% at 24 h, 48 h, and 72 h, respectively. As shown in Fig. 4C, F. The other percentages for siRNAs alone are detailed in the supplementary file (Table S2, S3, and S4).

### Gene expression profiling for human cell death pathway

#### Effect of siPFKFB4 on gene expression in U87-MG cells

**Fig. 5:**
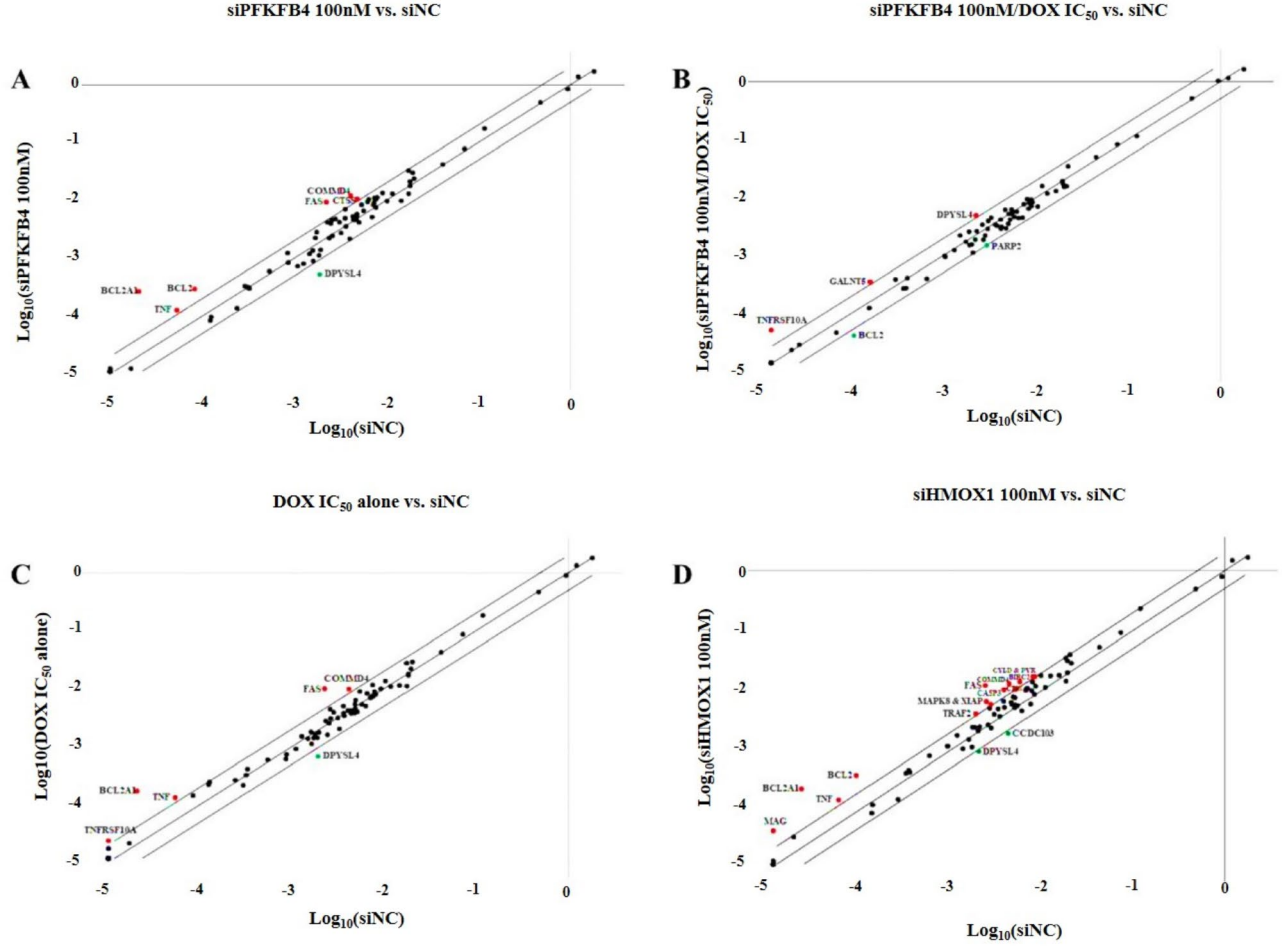
(**A**) The scatter plot illustrates the changes in gene expression for the siPFKFB4 100 nM treatment group, with up-regulated genes marked by red dots and down-regulated genes represented by green dots. (**B**) Another scatter plot highlights the gene expression alterations observed in the siPFKFB4 100 nM combined with the DOX IC_50_ group, employing the same color scheme for clarity. (**C**) This plot depicts gene expression patterns for the DOX IC_50_ group, once more using red dots for up-regulated genes and green dots for those that are down-regulated. (**D**) Finally, the scatter plot for the siHMOX1 100 nM group shows gene expression changes, with up-regulated genes indicated in red and down-regulated genes in green, specifically highlighting those with a fold change of ≥ 2 and a *p*-value of < 0.05.

To identify genes associated with apoptosis, we performed a PCR array as detailed in the Materials and Methods section, assessing the influence of siPFKFB4 on 84 genes within U87-MG cells. This analysis targeted the changes in gene expression related to glioblastoma following treatment with 100 nM siPFKFB4 for 48 h. This specific concentration was selected based on our cell cytotoxicity assay results, as it is higher than the IC_50_ value for siPFKFB4 in U87-MG cells after 72 h of exposure. Using a 2-fold change as a standard cutoff to ensure data reliability and highlight significant expression shifts, the analysis identified notable alterations in seven genes, with six showing marked upregulation and one demonstrating downregulation, all with a fold change of ≥ 2 and a statistically significant *p*-value (*p* < 0.05). As shown in Fig. 5A and Table 2.

**Table 2:** Up-regulated genes (A) and down-regulated genes (B) in U87-MG treated with siPFKFB4 100nM.

A			B		
Gene Symbol	Fold Regulation	GO	Gene Symbol	Fold Regulation	GO
BCL2	3.54	Anti-apoptotic	DPYSL4	− 3.56	Regulates apoptosis
BCL2A1	12.95	Anti-apoptotic			
COMMD4	2.94	Repair of DNA			
CTSS	2.17	Regulates apoptosis			
FAS	4.17	Regulates apoptosis			
TNF	2.39	Regulates apoptosis			

GO: Gene Ontology.

#### Effect of siPFKFB4/DOX on gene expression in U87-MG cells

To investigate the genes involved in apoptosis, we performed a PCR array, as described in the Materials and Methods section, to assess the combined effects of siPFKFB4 and DOX on the expression of 84 genes in U87-MG cells. This was done to identify any expression changes in genes associated with glioblastoma after treatment with 100 nM siPFKFB4 plus DOX at its IC_50_ concentration for a duration of 48 h. These specific concentrations were selected based on findings from our cell cytotoxicity assay, which indicated that they surpass the IC_50_ values for both siPFKFB4 and DOX in U87-MG cells after 72 h of exposure. We applied a 2-fold change as a standard cut-off to filter the data, ensuring only significant expression changes were captured. The analysis demonstrated that out of the 84 genes examined, three genes were markedly upregulated, while another three were downregulated, all exhibiting a fold change of ≥ 2 and achieving a statistically significant p-value (*p* < 0.05). As shown in Fig. 5B and Table S5.

#### Effect of DOX alone on gene expression in U87-MG cells

To identify genes implicated in apoptosis, we conducted a PCR array following the protocol outlined in the Materials and Methods section, focusing on the effects of DOX treatment alone on the expression of 84 genes in U87-MG cells. This analysis aimed to capture expression changes in glioblastoma-related genes after administering DOX at its IC_50_ concentration over a 48-hour period. The selected concentration was informed by our cell cytotoxicity assay, which confirmed that it surpasses the IC_50_ value for DOX in U87-MG cells after 72 h. To ensure data reliability and isolate genes with substantial expression changes, we applied a 2-fold change as a standard cutoff. The results revealed significant upregulation in five genes, while only one gene showed downregulation, each with a fold change of ≥ 2 and a *p*-value below 0.05 As shown in Fig. 5C and Table S6.

#### Effect of siHMOX1 alone on gene expression in U87-MG cells

To investigate the influence of siHMOX1 on apoptosis-related genes, we carried out a PCR array, following the procedures detailed in the Materials and Methods section. This analysis targeted 84 genes within U87-MG cells, aiming to discern expression changes linked to glioblastoma after administering siHMOX1 (100 nM) for 48 h. This concentration was chosen since it surpasses the IC_50_ value of siHMOX1 in U87-MG cells as established by our cytotoxicity assay conducted over 72 h. To ensure that our findings were both robust and significant, a two-fold change in gene expression was employed as the threshold criterion. The results indicated a considerable shift in gene expression, with 14 genes showing notable upregulation and 2 genes exhibiting downregulation, each surpassing a fold change of 2 and achieving statistical significance (*p* < 0.05). As shown in Fig. 5D and Table S7.

### Bioinformatics analysis

#### Analysis of differential gene expression (DiffExp) by TIMER database

**Fig. 6:**
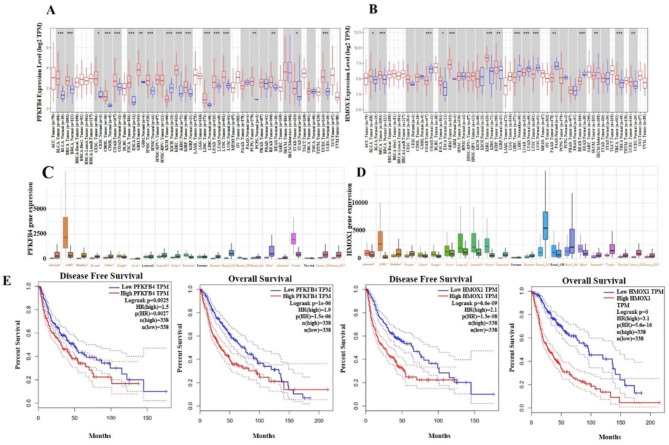
(**A**) Gene expression analysis of *PFKFB4* in all cancer cohorts of TCGA using TIMER database. (**B**) Gene expression analysis of *HMOX1* in all cancer cohorts of TCGA TIMER database. (**C**) Gene expression analysis of *PFKFB4* in using TNMplot server. (**D**) Gene expression analysis of *HMOX1* in using TNMplot server. (**E**) Kaplan-Meier plot for Disease-free survival (DFS) and overall survival (OS) analysis based on gene expression of *PFKFB4* and *HMOX1* by GEPIA web tool.

Gene expression analysis of *PFKFB4* and *HMOX1* was performed in all cancer cohorts of TCGA using the TIMER (Tumor Immune Estimation Resource) database. The results of TIMER analysis include a “DiffExp” module for analyzing gene expression differences in tumor and adjacent normal tissue. The results presented gene expression levels in box plots and determined differential expression using the Wilcoxon test. From the box plots we identified genes that are up- or down-regulated in the tumors (red box plots) compared to normal tissues (blue box plots) for each cancer type displayed in gray columns versus the normal data as shown in **(**Fig. 6A**)**. One asterisk (*) this indicates statistical significance in the gene’s expression of interest at the *p* < 0.05 level compared to what is in the normal tissues and so on. The two asterisks (**) typically indicates a higher level of significance, often at the *p* < 0.01 level. The three asterisks (***) represent an even higher level of significance, often at the *p* < 0.001 level. For example, statistically, *PFKFB4* was up-regulated in BLCA and BRCA tumors with three asterisks, up-regulated in PCPG and READ tumors with two asterisks, up-regulated in with CESC and STAD tumors with one asterisk, and with no significant difference in tumors as BRCA-Basal and BRCA-LumA tumors. For *HMOX1*, it was, for example, statistically up-regulated in BRCA and COAD tumors with three asterisks, up-regulated in KRIP and PAAD tumors with two asterisks, up-regulated in with BLCA and ESCA tumors with one asterisk, and with no significant difference in tumors as BRCA-Basal and BRCA-LumA tumors as shown in **(**Fig. 6B**)** .

#### Pan-cancer analysis by TNMplot server

Gene expression analysis for PFKFB4 and HMOX1 was performed using the TNMplot server. The findings are illustrated through boxplots, which display the differential expression of these two genes across most of the ten most common tumor types. Notable differences, determined by the Mann–Whitney U test, are marked in red (* *p* < 0.01). In the Boxpots the location of the median line inside the box indicates the central tendency of each group’s data. The spread of the data represents the size of the box and whiskers. Our pan-cancer analysis indicated a statistically significant expression of the PFKFB4 gene in various tumor types, including adrenal, acute myeloid leukemia (AML), bladder, breast, colon, esophageal, liver, lung squamous cell carcinoma, ovarian, pancreatic, rectal, renal clear cell carcinoma, renal chromophobe, renal papillary, skin, stomach, testicular, as well as in both endometrial and cervical uterine cancers. On the other hand, the gene expression of *PFKFB4* was not statistically significant in the following tumors: Lung-AC, Prostate, and Thyroid as shown in **(**Fig. 6C**)**. The gene expression of *HMOX1* was statistically significant in the following tumors: Adrenal, AML, Bladder, Breast, Colon, Esophageal, Liver, Lung-SC, Ovary, Pancreas, Rectum, Renal-CC, Renal-PA, Skin, Stomach, Testis, Thyroid, Uterus-CS, and Uterus-EC. On the other Hand, the *HMOX1* expression was not statistically significant in Prostate and Renal-CH. The difference in statistics might or might not affect the biology or pathology being studied as shown in **(**Fig. 6D**)** .

#### GEPIA (Gene expression profiling and interactive analyses)

GEPIA web tool analysis was performed to generate the Kaplan-Meier plot for overall survival (OS) and the disease-free survival (DFS) outcomes based on gene expression for *PFKFB4* and HMOX1 to gain insights into the relationship between gene expression and survival outcomes. The x-axis in the Kaplan-Meier plot represents time in months, while the y-axis represents the probability of survival or remaining disease-free. There are two main curves, each representing a different group; high expression in red color vs. low expression of the gene in blue color). Four Kaplan-Meier plots were generated; two of them for overall survival (OS) and the disease-free survival (DFS) outcomes for *PFKFB4* and the two for *HMOX1*. The results showed the survival blue curves for the low-expression group in all the above-mentioned plots were higher which suggests that lower gene expression is associated with better survival and longer disease-free period. GEPIA provides statistical information such as hazard ratios and p-values, suggests statistical significance. A hazard ratio less than 1 indicates a protective effect (higher gene expression associated with better survival), while a hazard ratio greater than 1 indicates a risk factor (lower gene expression associated with better survival). Our results showed that the hazard ratios in all the four Kaplan-Meier plots are greater than 1 which indicate that the risk factor of lower gene expression for *PFKFB4* and *HMOX1* are associated with better survival overall survival (OS) and disease-free survival (DFS) outcomes as shown in **(**Fig. 6E**)**. No previous studies have explicitly investigated the biological link between *PFKFB4* and *HMOX1* gene expressions using the GEPIA database, highlighting a gap in the current literature on their combined role in cancer survival outcomes.

#### STRING protein-protein interaction networks functional enrichment analysis

**Fig. 7:**
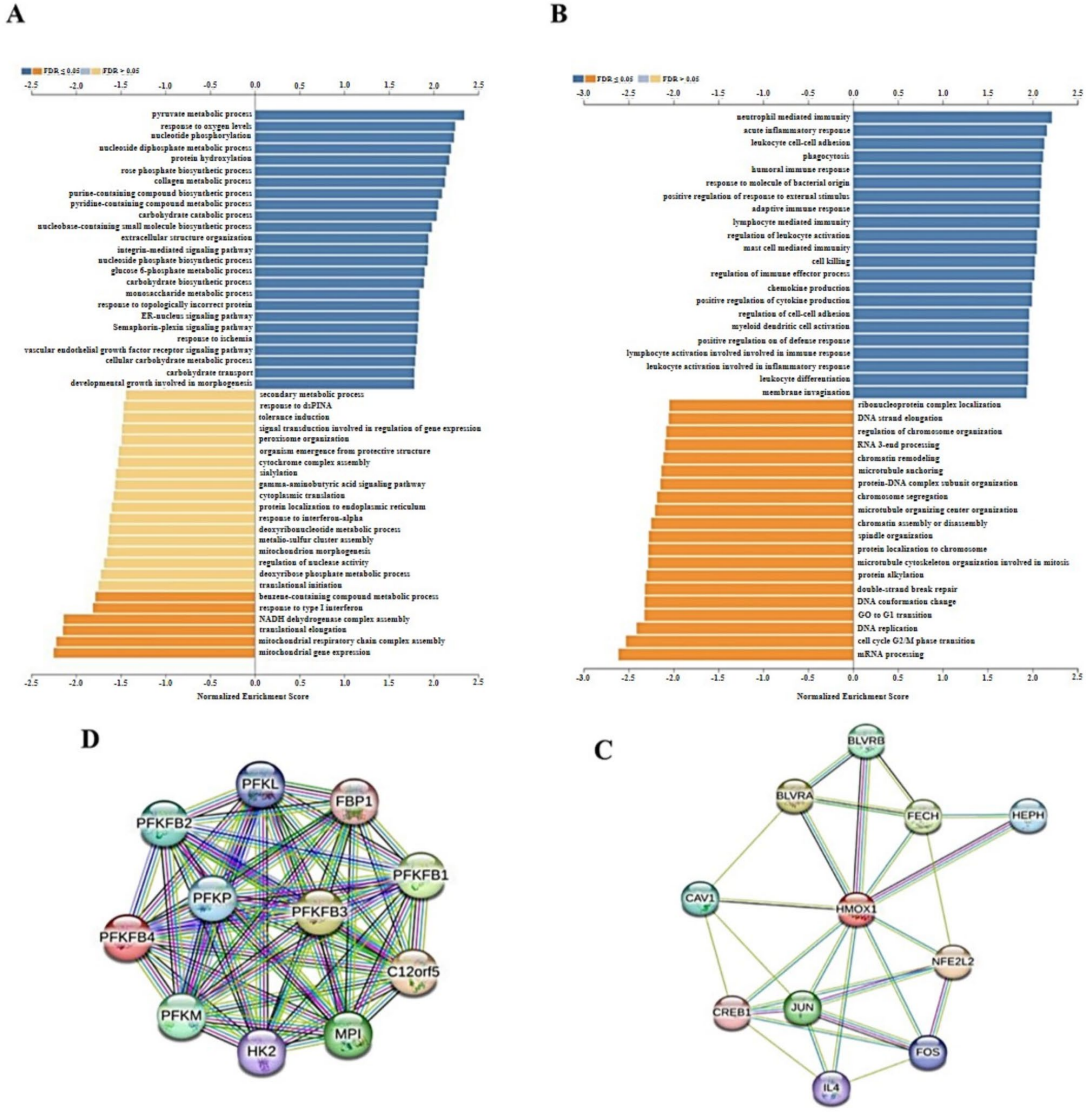
Positively and negatively Co-expressed genes biological processes enrichment. (**A**) *PFKFB4* positively and negatively Co-expressed genes biological processes enrichment. (**B**) *HMOX1* positively and negatively Co-expressed genes biological processes enrichment. (**C**) *PFKFB4* PPi. (**D**) *HMOX1* PPi.

The co-expressed genes biological processes (normalized enrichment score analysis) was performed for *PFKFB4* and HMOX1 using the STRING database with False Discovery Rate (FDR) threshold at 0.05 as shown in Fig. 7A, and B. The analysis revealed positively and negatively co-expressed genes biological processes enrichment for both *PFKFB4* and *HMOX1*. The protein-protein interaction (PPI) analysis was conducted for PFKFB4 and HMOX1 using the STRING database. The analysis revealed the PPI network for both genes as shown in Fig. 7D, and C^24^.

## Discussion

This study examined the anti-proliferative effects of siPFKFB4 and siHMOX1, with doxorubicin (DOX) and temozolomide (TMZ), on glioblastoma cells (U87-MG). Under normoxic conditions, glioblastoma cells exhibited higher levels of PFKFB4 and HMOX1 compared to normal fibroblast cells (HDFa). PFKFB4 is vital in glucose metabolism in cancer cells, converting fructose-2,6-bisphosphate to fructose-6-phosphate, a crucial step in glycolysis. Its role in regulating the cell cycle and apoptosis makes PFKFB4 a promising target for therapy^9^. Studies suggest that inhibiting PFKFB4 disrupts glucose metabolism in cancer cells, offering a potential treatment strategy for glioblastoma^25^.

HMOX1, an enzyme essential for heme breakdown, is involved in the progression of several cancers, including glioblastoma. HMOX1 supports rapid tumor growth, cancer aggressiveness, angiogenesis, and metastasis^26^. Byproducts of HMOX1 also modulate the immune response, making it another therapeutic target. Suppressing HMOX1 has been shown to enhance the effectiveness of chemotherapeutic agents like doxorubicin in breast cancer and TMZ in melanoma^27^. This is consistent with studies showing that PFKFB4 and HMOX1 are overexpressed in various cancer types^12^ and are linked to poorer prognosis and reduced survival rates in brain cancer patients^13^.

Our findings indicate that transfecting U87-MG cells with siPFKFB4 and siHMOX1 inhibited cell viability compared to scramble transfection. This aligns with other studies suggesting that knocking down PFKFB4 and HMOX1 induces apoptosis and suppresses cancer cell growth^25^. Zhao et al.^28^ and Liu et al.^29^ also reported that siRNAs blocking PFKFB4 and HMOX1 inhibit glioblastoma cell proliferation. Although siPFKFB4 and siHMOX1 reduced mRNA levels of their targets, DOX alone did not affect PFKFB4 mRNA expression. Silencing PFKFB4 and HMOX1 reduces ROS detoxification via glycolysis, inhibiting cell growth and movement^30^. Clem and Telang^31^ found that these siRNAs do not have anti-proliferative effects on normal cells (HDFa) as they do on U87-MG cells.

Future research should investigate how knocking down HMOX1 and PFKFB4 affects drug resistance pathways in glioblastoma, such as PI3K/AKT, MAPK, and NF-κB. Using inhibitors specific to these pathways could provide deeper insights into the molecular mechanisms at play.

Glioblastoma cells are known for their resistance to anti-tumor drugs, making brain tumor treatment challenging. This study aimed to enhance the cytotoxicity of DOX and TMZ against glioblastoma cells by targeting PFKFB4 and HMOX1^32^. Our results showed that RNA interference-mediated silencing of PFKFB4 and HMOX1, combined with DOX and TMZ, had a more potent effect on cell proliferation and migration. siPFKFB4 enhanced DOX’s cytotoxicity by lowering its IC_50_ but had a lesser effect with TMZ. Conversely, siHMOX1 inhibited cell migration but did not synergize with DOX to suppress cell proliferation, consistent with Zhu et al.^33^, who observed that DOX-induced HMOX1 overexpression led to resistance in U87-MG cells. siHMOX1 had a mild effect with TMZ on U87-MG cell proliferation but decreased cell migration. HMOX1 supports tumor angiogenesis by increasing VEGF expression^34^, and its blockade might inhibit tumor progression^26^. Long-term studies are needed to assess the chronic efficacy and safety of targeting HMOX1 and PFKFB4 in glioblastoma models.

We also investigated the role of PFKFB4 and HMOX1 in glioblastoma growth by examining death-associated genes in U87-MG cells. siPFKFB4 alone upregulated several apoptotic genes, such as BCL2, BCL2A1, COMMD4, CTSS, FAS, and TNF. Overexpression of BCL-2 in glioma cells is known to evade apoptosis, contributing to drug resistance^35^. Post-siRNA treatment, glioblastoma cells showed resistance to TMZ due to upregulated BCL-2 and TOPO-2, which inhibit apoptosis through NF-kappa B activation (81–84). Despite XIAP and BCL-2 being anti-apoptotic, blocking them is linked to increased apoptosis and reduced glioma survival^36^. Both BCL-2 and XIAP can suppress apoptosis by regulating TRAIL and caspase-3-mediated cell death^37^. Comprehensive toxicity profiling is crucial to evaluate the impact of gene knockdown on non-tumor tissues and organs. This can be achieved through histopathology and serum biomarker analysis to assess systemic toxicity, ensuring that treatments targeting PFKFB4 and HMOX1 are safe for clinical use.

Our findings, supported by Wang, Shi et al.^38^, showed that blocking BCL-2 in glioma cells post-mRNA blockade therapy led to tumor-suppressing activities of the MAPK/ERK pathway. Antisense BCL-2 expression significantly increased cisplatin cytotoxicity and reduced glioma cell survival^39^. A significant change observed post-siPFKFB4 100 nM/DOX IC_50_ exposure was BCL-2 downregulation, explaining the reduction in doxorubicin IC_50_ concentration in U87-MG cells treated with siPFKFB4 alone. Concurrently, upregulation of BCL-2 and XIAP decreased cell proliferation and apoptosis in the siHMOX1 group, accompanied by reduced levels of caspase-3. TRAF2 upregulation promotes tumor progression and invasion^40^. Silencing TRAF2 reduces glioblastoma growth^41^, justifying the lower effect of siHMOX1 compared to siPFKFB4 and siPFKFB4/DOX. To ensure the broad applicability of our results, it is necessary to validate findings in glioblastoma models representing different genetic backgrounds, including IDH1-mutant and IDH1-wildtype glioblastomas.

BCL2A1, an anti-apoptotic protein, is not primarily associated with brain cancer metastases but plays a role in lymphoid and myeloid leukemia development^42^. Overexpression of BCL2A1 enhances tumor cell survival and resistance to pro-apoptotic agents like TNF-α^42^. In our study, BCL2A1 was upregulated in siPFKFB4, siHMOX1, and DOX alone groups but not in the siPFKFB4 100 nM/DOX IC_50_ group. Previous studies linked silencing of anti-apoptotic genes to increased chemotherapy effectiveness. Silencing BCL2A1 in prostate cancer decreased cell count, increased apoptosis, and improved cabazitaxel resistance^43^.

COMMD4 expression is associated with poor prognosis in several cancers, including NSCLC and glioblastoma, influencing tumor proliferation, invasion, metastasis, and drug resistance^44^. Silencing COMMD4 decreases cell viability and proliferation^45^. In this study, COMMD4 expression was upregulated in siPFKFB4, siHMOX1, and DOX alone groups, explaining glioblastoma cells’ resistance to treatment.

Cathepsin S (CTSS), a cysteine protease, induces autophagy and mitochondrial apoptosis in glioblastoma cells; its blockade by siRNA lowers apoptosis-mediated cathepsin S process^46^. CTSS expression is higher in patients with glioblastoma, breast cancer, and papillary thyroid cancer, associated with poorer prognosis^47^. Suppression of CTSS significantly reduced tumor metastasis^48^. The CTSS gene was upregulated in siPFKFB4 and siHMOX1 alone groups, explaining higher proliferation and cell viability compared to the siPFKFB4 100 nM/DOX IC_50_ group.

Fas (CD95/APO-1) is a cell surface “death receptor” promoting apoptosis-mediated caspase-3 cascade in tumor cells when engaged by FasL ligand. If apoptosis is impaired, Fas/FasL signaling can promote tumor invasion^49^. The FAS pathway is overexpressed in high-grade gliomas (HGG)^50^, stimulating glioblastoma proliferation and survival independent of caspase by promoting cytokines and chemokines (IL-6, IL-8, and MCP-1^51^. Inducing apoptosis through Fas/Fas ligand (Fas-L)-related immunotherapy is possible^52^. In this study, the presence of FAS in siPFKFB4 100 nM, siHMOX1, and DOX alone groups displayed less programmed cell death compared to the loss of expression in the siPFKFB4 100 nM/DOX IC_50_ group, indicating alternative apoptotic pathways exist apart from the Fas cascade.

High TNF-α expression in glioblastoma is linked to tumor progression and invasion^53^, and its silencing reduces resistance to inhibitors like EGFR inhibitors^54^. Similar to the Fas gene, high TNF-α expression in siPFKFB4 100 nM, siHMOX1, and DOX alone groups elaborates higher cell proliferation and viability compared to the siPFKFB4 100 nM and DOX combination group, demonstrating a higher apoptotic rate and lower cell growth.

TNFRSF10A is a cell surface receptor activating the extrinsic pathway of apoptosis in cancer cells when binding to tumor necrosis factor-related apoptosis-inducing ligands^55^. This gene was found only in groups with DOX treatment, with higher expression in the siPFKFB4 100 nM and DOX combination group than DOX alone, explaining enhanced chemotherapy cytotoxicity. Although PARP2 plays a role in the repair system by detecting DNA breaks and recruiting repair factors, the PARP2 protein promotes proliferation and invasion in cancers like prostate cancer^56^. Priya et al. (2022) found that silencing PARP-2 or PARP-1 alone stimulated the immune response in tumor microenvironments^57^. PARP silencing can enhance the potency of TMZ^57^. Inhibiting PARP2 in siPFKFB4 100 nM, siHMOX1, and DOX alone, and its downregulation in siPFKFB4 100 nM and DOX, led to suppressed cell proliferation and improved chemotherapy potency against aggressive tumor cells.

The CCDC103 gene plays a crucial role in cancer growth^58^. Xu and Chen^58^reported high CCDC103 expression in glioma, with patients having worse prognosis and suggested it as a new prognostic marker in glioma. Knockdown of CCDC103 mRNA dramatically inhibited glioma cell growth and migration^58^. The CCDC103 gene was downregulated by siHMOX1 treatment, contributing to reduced U87-MG cell proliferation and apoptosis.

DPYSL4 induces apoptosis in response to DNA damage through p53 regulation and is linked to cancer invasion and progression^59^. Low DPYSL4 expression is associated with poor survival in breast and ovarian cancers. DPYSL4 is highly expressed in glioma cells to regulate neuronal differentiation and apoptosis^60^. DPYSL4 was exclusively upregulated in the siPFKBF4 100 nM/DOX group as an apoptotic agent compared to downregulation in other monotherapy groups, indicating synergistic effects of siRNA and chemotherapy against tumor growth and invasion.

The overexpression of the BIRC2 gene may promote tumor growth in U87-MG through siHMOX1 treatment. Debangshu et al. (2020) showed that BIRC2 regulates NF-κB, crucial for tumor development by blocking apoptosis^61^. Cylindromatosis (CYLD) gene knockdown promotes apoptosis resistance, tumor progression, and development^62^. CYLD overexpression in the siHMOX1 group may induce apoptosis.

Our study showed an increase in MAPK8, PVR, and MAG gene expression in the siHMOX1 group. Activating the MAPK signaling pathway by MAPK8 in glioblastoma cells accelerates proliferation, inhibits apoptosis, and promotes resistance to TMZ^63^. High expression of PVR and MAG genes in U87-MG cells is associated with poor prognosis due to their role as cell adhesion molecules promoting tumor growth and metastasis^64^. These data are compatible with our gene expression results in the siHMOX1 group.

The GALNT5 gene, part of the GALNT family, enhances EGF/EGFR activation in cancers like cholangiocarcinoma, hepatocellular carcinoma, and ovarian cancer^65^. Silencing GALNT5 reduces tumor proliferation, migration, and invasion^66^. Although treatment with both siPFKFB4 100 nM and DOX was the highest compared to others, the GALNT5 gene was upregulated, and its role remains unclear in this group. Further studies are required.

We discovered that PFKFB4 and HMOX1 control glioma’s growth, migration, invasion, and malignancy by studying apoptotic genes and others. Modulating the glioma chemoresistant phenotype by inhibiting PFKFB4 and HMOX1 may enhance TMZ or DOX therapy.

Our findings revealed significant differences in gene expression patterns for PFKFB4 and HMOX1 across various cancer types. PFKFB4 exhibited substantial upregulation in bladder cancer (BLCA) and breast cancer (BRCA) with high statistical significance. It was also significantly upregulated in cancers like PCPG and READ, with moderate significance, and had low significance in cancers like CESC and STAD. No significant difference was observed in BRCA-Basal and BRCA-LumA tumors.

For HMOX1, analysis revealed statistically significant gene expression in a wide range of tumors, such as adrenal, AML, bladder, breast, colon, esophageal, liver, lung-SC, ovary, pancreas, rectum, renal-CC, renal-PA, skin, stomach, testis, thyroid, uterus-CS, and uterus-EC. However, no significant differences were seen in prostate and renal-CH tumors. These variations in statistical significance may or may not have implications for the underlying biology or pathology. Our results consistently showed higher survival curves for the low-expression group, indicating lower gene expression was associated with improved survival and a longer disease-free period. Hazard ratios greater than 1 across all Kaplan-Meier plots underlined the risk factor associated with lower gene expression for both PFKFB4 and HMOX1 in terms of OS and DFS outcomes. In conclusion, these findings collectively highlight the differential gene expression and potential clinical significance of PFKFB4 and HMOX1. A notable limitation of this study is the lack of protein-level validation for siRNA knockdown using Western blot analysis. While qPCR provides reliable insights into mRNA expression changes, confirming these effects at the protein level is crucial for a comprehensive understanding of the knockdown’s impact. Future studies should incorporate Western blotting or other protein-level assessments to strengthen these findings and provide further mechanistic insights. Additionally, this study demonstrates significant changes in gene expression associated with chemosensitization and apoptosis; however, a limitation of the PCR array approach is its inability to fully elucidate the underlying molecular mechanisms. To address this, future research will utilize RNA sequencing under similar experimental conditions to provide comprehensive insights into pathways such as ROS generation, mitochondrial dysfunction, and other cellular processes involved in chemosensitization. Incorporating these mechanistic studies will enhance our understanding of how siRNA treatment potentiates the effects of chemotherapeutic agents. Future studies should prioritize incorporating Western blotting to strengthen these findings. While this study demonstrates significant changes in gene expression associated with chemosensitization and apoptosis, a limitation of the PCR array approach is its inability to fully elucidate the underlying molecular mechanisms. To address this, future research will utilize RNA sequencing under similar experimental conditions to provide comprehensive insights into pathways such as ROS generation, mitochondrial dysfunction, and other cellular processes involved in chemosensitization. Incorporating these mechanistic studies will enhance our understanding of how siRNA treatment potentiates the effects of chemotherapeutic agents.” While this study demonstrates significant changes in gene expression associated with chemosensitization and apoptosis, a limitation of the PCR array approach is its inability to fully elucidate the underlying molecular mechanisms. To address this, future research will utilize RNA sequencing under similar experimental conditions to provide comprehensive insights into pathways such as ROS generation, mitochondrial dysfunction, and other cellular processes involved in chemosensitization. Incorporating these mechanistic studies will enhance our understanding of how siRNA treatment potentiates the effects of chemotherapeutic agents.

## Conclusion

In conclusion, we found that *PFKFB4* silencing decreased In vitro U87-MG glioblastoma cell growth. In addition, *PFKFB4* blockage potentiates DOX-induced glioblastoma cytotoxicity with low toxicity on normal cells. On the other hand, the *HMOX1* blockade exhibited a mild effect with TMZ and no effect with DOX against U87-MG cell proliferation, but decreased cell migration In vitro. Also, our data show that reduced PFKFB4 levels in U87-MG glioblastoma cells enhance the anti-angiogenic effect of DOX and TMZ. Additionally, the combination treatment with siPFKFB4/DOX causes alteration in different genes that may work towards the glioblastoma cells to suicide. In addition, this study suggests that the down-regulation of *BCL2* and *PARP2* as well as up-regulation of *TNFRSF10A* expression may be responsible for DOX-induced U87-MG cell apoptosis. However, further research and much work should be done to develop a formulation that both improves the bioavailability of medications to the central nervous system (CNS) and allows for a less invasive route of administration in the future. Due to limited resources, protein-level validation of PFKFB4 and HMOX1 knockdown using Western blot was not feasible. This aspect will be prioritized in subsequent studies to complement the current findings.

## Electronic supplementary material

Below is the link to the electronic supplementary material.

Supplementary Material 1

Supplementary Material 2

## Data Availability

All data generated or analyzed during this study are included in this published article.

## References

[1] Obrador, E. et al. Glioblastoma therapy: past, present and future. *Int. J. Mol. Sci.* **25**, 2529 (2024). 38473776 10.3390/ijms25052529 PMC10931797

[2] Venkataramani, V. et al. Disconnecting multicellular networks in brain tumours. *Nat. Rev. Cancer*. **22**, 481–491 (2022). 35488036 10.1038/s41568-022-00475-0

[3] Shergalis, A., Bankhead, A., Luesakul, U., Muangsin, N. & Neamati, N. Current challenges and opportunities in treating glioblastoma. *Pharmacol. Rev.* **70**, 412–445 (2018). 29669750 10.1124/pr.117.014944 PMC5907910

[4] Miyauchi, J. T. & Tsirka, S. E. Advances in immunotherapeutic research for glioma therapy. *J. Neurol.* **265**, 741–756 (2018). 29209782 10.1007/s00415-017-8695-5 PMC5914508

[5] Pridham, K. J. et al. PIK3CB/p110β is a selective survival factor for glioblastoma. *Neuro Oncol.* **20**, 494–505 (2018). 29016844 10.1093/neuonc/nox181 PMC5909664

[6] Elshaer, S. S. et al. MiRNAs role in glioblastoma pathogenesis and targeted therapy: signaling pathways interplay. *Pathol Oncol Res*. **154511** (2023). 10.1016/j.prp.2023.154511 37178618

[7] Polivka Jr, J. & Janku, F. Molecular targets for cancer therapy in the PI3K/AKT/mTOR pathway. *InPharm Association.* **142**, 164–175 (2014). 24333502 10.1016/j.pharmthera.2013.12.004

[8] Li, X. et al. PI3K/Akt/mTOR signaling pathway and targeted therapy for glioblastoma. *Oncotarget*. **7**, 33440 (2016). 26967052 10.18632/oncotarget.7961 PMC5078108

[9] Cai, Y. C. et al. PFKFB4 overexpression facilitates proliferation by promoting the G1/S Transition and is associated with a poor prognosis in triple-negative breast cancer. *Dis Markers.* (2021). (2021). 10.1155/2021/8824589 PMC8211511 34211613

[10] Yan, S. et al. PFKFB3 Inhibition attenuates oxaliplatin-induced autophagy and enhances its cytotoxicity in colon cancer cells. *Int. J. Mol. Sci.* **20**, 5415 (2019). 31671668 10.3390/ijms20215415 PMC6862230

[11] Fang, J., Akaike, T. & Maeda, H. Antiapoptotic role of Heme Oxygenase (HO) and the potential of HO as a target in anticancer treatment. *Apoptosis. ***9**, 27–35 (2004). 14739596 10.1023/B:APPT.0000012119.83734.4e

[12] Alfreahat, I. et al. In Vitro Potentiation of Doxorubicin Cytotoxicity Utilizing Clarithromycin Loaded-PEGylated Liposomes. *Technol. Cancer Res. Treat*. **24**, 15330338241312561 (2025). 10.1177/15330338241312561 PMC11770720 39865928

[13] Zeilstra, J. et al. Stem cell CD44v isoforms promote intestinal cancer formation in apc (min) mice downstream of Wnt signaling. *Oncogene.* **33**, 665–670 (2014). 23318432 10.1038/onc.2012.611

[14] Al-Awaida, W., Al-Ameer, H. J., Sharab, A. & Akasheh, R. T. Modulation of wheatgrass (Triticum aestivum Linn) toxicity against breast cancer cell lines by simulated microgravity. *Curr Res Toxicol.* **5**, 100127. 10.1016/j.crtox.2023.100127 (2023). 37767028 10.1016/j.crtox.2023.100127 PMC10520342

[15] Li, W. et al. The role of CD44 in glucose metabolism in prostatic small cell neuroendocrine carcinoma. *Mol Cancer Res*. **14**, 344–353 (2016). 10.1158/1541-7786.MCR-15-0466 PMC4834240 26832214

[16] Al Hanjori, A. S., Alshaer, W., Al-Anati, B., Wehaibi, S. & Zihlif, M. J. Studying the Anti-Tumor effects of SiRNA gene Silencing of some metabolic genes in pancreatic ductal adenocarcinoma. *Curr Mol Pharmacol*. **14**, 604–619 (2021). 10.2174/1874467213666201012162250 33045974

[17] Gallego-Yerga, L., Chiliquinga, A. J. & Peláez, R. J. I. J. o. M. S. Novel Tetrazole Derivatives Targeting Tubulin Endowed with Antiproliferative Activity against Glioblastoma Cells. *Int J Mol Sci*. **24**, 11093 (2023). 10.3390/ijms241311093 PMC10342533 37446273

[18] Al Azzam, K. M. et al. Assessment of the anticancer potential of certain phenolic and flavonoid components in ginger capsules using colorectal cancer cell lines coupled with quantitative analysis. *Biomed. Chromatogr*. **38**, e5993 (2024). 10.1002/bmc.5993 39152776

[19] Jing, S. et al. Levistilide a induces ferroptosis by activating the Nrf2/HO-1 signaling pathway in breast cancer cells. *Drug Des Devel Ther.* 2981–2993 (2022). 10.2147/DDDT.S374328 PMC9464640 36105321

[20] Guo, C. et al. PPA1 promotes breast cancer proliferation and metastasis through Pi3K/AKT/GSK3β signaling pathway. *Front Cell Dev Biol*. **9**, 730558 (2021). 10.3389/fcell.2021.730558 PMC8476924 34595179

[21] Kgosana, M. R. et al. Exploring the wound healing potential of lobostemon fruticosus using in vitro and in vivo bioassays. *J. Ethnopharmacol*. 336, 118632 (2025). 10.1016/j.jep.2024.118632 39069028

[22] Kanehisa, M., Furumichi, M., Sato, Y., Matsuura, Y. & Ishiguro-Watanabe, M. KEGG: biological systems database as a model of the real world. *Nucleic Acids Res*. **53**, D672–D677 (2025). 10.1093/nar/gkae909 PMC11701520 39417505

[23] Liu, T. et al. Identification of genes and pathways potentially related to PHF20 by gene expression profile analysis of glioblastoma U87 cell line. *Cancer Cell Int*. **17**, 1–12 (2017). 10.1186/s12935-017-0459-x PMC5628484 29033691

[24] Szklarczyk, D. et al. STRING v11: protein–protein association networks with increased coverage, supporting functional discovery in genome-wide experimental datasets. *Nucleic Acids Res*. **47**, D607–D613 (2019). 10.1093/nar/gky1131 PMC6323986 30476243

[25] Li, X. et al. The metabolic role of PFKFB4 in androgen-independent growth in vitro and PFKFB4 expression in human prostate cancer tissue. *BMC Urol.* **20**, 1–8 (2020). 32487245 10.1186/s12894-020-00635-0 PMC7268689

[26] Zerfaoui, M. et al. Nuclear localization of BRAFV600E is associated with HMOX-1 upregulation and aggressive behavior of melanoma cells. *Cancers (Basel)*. **14**, 311 (2022). 35053476 10.3390/cancers14020311 PMC8773521

[27] Sferrazzo, G. et al. Heme oxygenase-1 in central nervous system malignancies. *J Clin Med*. 9, 1562 (2020). 10.3390/jcm9051562 PMC7290325 32455831

[28] Zhao, K. et al. 6-Phosphofructo-2-kinase/fructose-2, 6-biphosphatase 4 acts as a protein kinase to regulate glioblastoma progression by activating the AKT/forkhead box O1 pathway. *Acta Biochim Pol.* **69**, 165–172 (2022). 35143148 10.18388/abp.2020_5789

[29] Lyu, W., Jia, B., Liu, W., Guo, Q. & Cathepsin S (CTSS) is highly expressed in temozolomide-resistant glioblastoma T98G cells and associated with poor prognosis. *Chinese*. *J. Cell. Mol. Immunol.* **36**, 924–929 (2020). 33148388

[30] Chiang, S. K., Chen, S. E. & Chang, L. C. A dual role of Heme oxygenase-1 in cancer cells. *Int. J. Mol. Sci.* **20**, 39 (2018). 30583467 10.3390/ijms20010039 PMC6337503

[31] Clem, B. et al. Small-molecule Inhibition of 6-phosphofructo-2-kinase activity suppresses glycolytic flux and tumor growth. *Mol. Cancer Ther.* **7**, 110–120 (2008). 18202014 10.1158/1535-7163.MCT-07-0482

[32] Azambuja, J. et al. CD73 as a target to improve Temozolomide chemotherapy effect in glioblastoma preclinical model. *Cancer Chemother. Pharmacol.* **85**, 1177–1182 (2020). 32417936 10.1007/s00280-020-04077-1

[33] Zhu, X. F. et al. Knockdown of Heme oxygenase-1 promotes apoptosis and autophagy and enhances the cytotoxicity of doxorubicin in breast cancer cells. *Oncol. Lett.* **10**, 2974–2980 (2015). 26722274 10.3892/ol.2015.3735 PMC4665608

[34] Birrane, G., Li, H., Yang, S., Tachado, S. D. & Seng, S. Cigarette smoke induces nuclear translocation of Heme Oxygenase 1 (HO-1) in prostate cancer cells: nuclear HO-1 promotes vascular endothelial growth factor secretion. *Int. J. Oncol.* **42**, 1919–1928 (2013). 23591596 10.3892/ijo.2013.1910 PMC3699615

[35] Teng, H., Li, M., Qian, L., Yang, H. & Pang, M. Long non–coding RNA SNHG16 inhibits the oxygen–glucose deprivation and reoxygenation–induced apoptosis in human brain microvascular endothelial cells by regulating miR–15a–5p/bcl–2. *Mol. Med. Rep.* **22**, 2685–2694 (2020). 32945414 10.3892/mmr.2020.11385 PMC7453539

[36] Siegelin, M., Gaiser, T., Habel, A. & Siegelin, Y. Daidzein overcomes TRAIL-resistance in malignant glioma cells by modulating the expression of the intrinsic apoptotic inhibitor, bcl-2. *Neurosci. Lett.* **454**, 223–228 (2009). 19429088 10.1016/j.neulet.2009.03.031

[37] Doucette, T. et al. Bcl-2 promotes malignant progression in a PDGF‐B‐dependent murine model of oligodendroglioma. *Int. J. Cancer*. **129**, 2093–2103 (2011). 21171016 10.1002/ijc.25869 PMC3196817

[38] Wang, X. F. et al. MiR-181d acts as a tumor suppressor in glioma by targeting K-ras and Bcl-2. *J. Cancer Res. Clin. Oncol.* **138**, 573–584 (2012). 22207524 10.1007/s00432-011-1114-x PMC11824363

[39] Zhu, C. J., Li, Y. B. & Wong, M. C. Expression of antisense bcl-2 cDNA abolishes tumorigenicity and enhances chemosensitivity of human malignant glioma cells. *J. Neurosci. Res.* **74**, 60–66 (2003). 13130506 10.1002/jnr.10722

[40] Wei, B. et al. Knockdown of TNF receptor-associated factor 2 (TRAF2) modulates in vitro growth of TRAIL-treated prostate cancer cells. *Biomed. Pharmacother.* **93**, 462–469 (2017). 28667915 10.1016/j.biopha.2017.05.145

[41] Zheng, M. et al. Growth Inhibition and radiosensitization of glioblastoma and lung cancer cells by small interfering RNA Silencing of tumor necrosis factor receptor–associated factor 2. *Cancer Res.* **68**, 7570–7578 (2008). 18794145 10.1158/0008-5472.CAN-08-0632 PMC2597026

[42] Cruz-Muñoz, W. et al. Roles for endothelin receptor B and BCL2A1 in spontaneous CNS metastasis of melanomagenes relevant to spontaneous CNS melanoma metastasis. *Cancer Res.* **72**, 4909–4919 (2012). 22865454 10.1158/0008-5472.CAN-12-2194 PMC4334445

[43] Pucci, P. et al. LncRNA HORAS5 promotes taxane resistance in castration-resistant prostate cancer via a BCL2A1-dependent mechanism. *Epigenomics.* **12**, 1123–1138 (2020). 32618200 10.2217/epi-2019-0316

[44] You, G. et al. COMMD proteins function and their regulating roles in tumors. *Front. Oncol.* 13 (2023). 10.3389/fonc.2023.1067234 PMC9910083 36776284

[45] Liu, Z. et al. COMMD4 is a novel prognostic biomarker and relates to potential drug resistance mechanism in glioma. *Novel Therapeutic Mech. Target. neuro-immune Regul. Neurol. Disorders*. **16648714**, 179 (2023).

[46] Zhang, L., Wang, H., Xu, J., Zhu, J. & Ding, K. Inhibition of cathepsin S induces autophagy and apoptosis in human glioblastoma cell lines through ROS-mediated PI3K/AKT/mTOR/p70S6K and JNK signaling pathways. *Toxicol. Lett.* **228**, 248–259 (2014). 24875536 10.1016/j.toxlet.2014.05.015

[47] Tan, J. et al. Integrated bioinformatics analysis reveals that the expression of cathepsin S is associated with lymph node metastasis and poor prognosis in papillary thyroid cancer. *Oncol. Rep.* **40**, 111–122 (2018). 29749483 10.3892/or.2018.6428 PMC6059735

[48] Sevenich, L. et al. Analysis of tumour-and stroma-supplied proteolytic networks reveals a brain-metastasis-promoting role for cathepsin S. *Nat. Cell Biol.* **16**, 876–888 (2014). 25086747 10.1038/ncb3011 PMC4249762

[49] Ge, Y., Yoshiie, K., Kuribayashi, F., Lin, M. & Rikihisa, Y. Anaplasma phagocytophilum inhibits human neutrophil apoptosis via upregulation of bfl-1, maintenance of mitochondrial membrane potential and prevention of caspase 3 activation. *Cell. Microbiol.* **7**, 29–38 (2005). 15617521 10.1111/j.1462-5822.2004.00427.x

[50] Werner, J. M. et al. Expression of FAS-L differs from primary to relapsed low-grade gliomas and predicts progression-free survival. *Anticancer Res.* **37**, 6639–6648 (2017). 29187439 10.21873/anticanres.12121

[51] Nat, R., Radu, E., Regalia, T. & Popescu, L. Apoptosis in human embryo development: 3. Fas-induced apoptosis in Brian primary cultures. *J. Cell. Mol. Med.* **5**, 417–428 (2001). 12067476 10.1111/j.1582-4934.2001.tb00177.x PMC6740267

[52] Tao, J., Qiu, B., Zhang, D. & Wang, Y. Expression levels of Fas/Fas-L mRNA in human brain glioma stem cells. *Mol. Med. Rep.* **5**, 1202–1206 (2012). 22344564 10.3892/mmr.2012.791

[53] Shamsdin, S. A., Mehrafshan, A., Rakei, S. M. & Mehrabani, D. Evaluation of VEGF, FGF and PDGF and serum levels of inflammatory cytokines in patients with glioma and meningioma in Southern Iran. *Asian Pac. J. Cancer Prevention: APJCP*. **20**, 2883 (2019). 10.31557/APJCP.2019.20.10.2883 PMC6982662 31653130

[54] Luo, Z., Wang, B., Liu, H. & Shi, L. TNF inhibitor Pomalidomide sensitizes glioblastoma cells to EGFR Inhibition. *Annals Clin. Lab. Sci.* **50**, 474–480 (2020). 32826244

[55] Li, T. et al. DDIT3 and KAT2A proteins regulate TNFRSF10A and TNFRSF10B expression in Endoplasmic reticulum stress-mediated apoptosis in human lung cancer cells. *J. Biol. Chem.* **290**, 11108–11118 (2015). 25770212 10.1074/jbc.M115.645333 PMC4409269

[56] Langelier, M. F., Lin, X., Zha, S. & Pascal, J. M. Clinical PARP inhibitors allosterically induce PARP2 retention on DNA. *Sci. Adv.* **9**, eadf7175 (2023). 36961901 10.1126/sciadv.adf7175 PMC10038340

[57] Bisht, P., Kumar, V. U., Pandey, R., Velayutham, R. & Kumar, N. Role of PARP inhibitors in glioblastoma and perceiving challenges as well as strategies for successful clinical development. *Front. Pharmacol.* **13** (2022). 10.3389/fphar.2022.939570 PMC9297740 35873570

[58] Zhu, X. et al. Novel biomarker genes for prognosis of survival and treatment of glioma. *Front. Oncol*. **11**, 667884 (2021). 10.3389/fonc.2021.667884 PMC8714878 34976783

[59] Kimura, J., Kudoh, T., Miki, Y. & Yoshida, K. Identification of dihydropyrimidinase-related protein 4 as a novel target of the p53 tumor suppressor in the apoptotic response to DNA damage. *Int. J. Cancer*. **128**, 1524–1531 (2011). 20499313 10.1002/ijc.25475

[60] Hooper, C. M., Hawes, S. M., Kees, U. R., Gottardo, N. G. & Dallas, P. B. Gene expression analyses of the spatio-temporal relationships of human Medulloblastoma subgroups during early human neurogenesis. *PLoS One*. **9**, e112909 (2014). 25412507 10.1371/journal.pone.0112909 PMC4239019

[61] Samanta, D. et al. BIRC2 expression impairs anti-cancer immunity and immunotherapy efficacy. *Cell. Rep.* **32**, 108073 (2020). 32846130 10.1016/j.celrep.2020.108073

[62] Brummelkamp, T. R., Nijman, S. M., Dirac, A. M. & Bernards, R. Loss of the cylindromatosis tumour suppressor inhibits apoptosis by activating NF-κB. *Nature* **424**, 797–801 (2003). 12917690 10.1038/nature01811

[63] Xu, P., Zhang, G., Hou, S. & Sha L.-g. MAPK8 mediates resistance to Temozolomide and apoptosis of glioblastoma cells through MAPK signaling pathway. *Biomed. Pharmacother.* **106**, 1419–1427 (2018). 30119215 10.1016/j.biopha.2018.06.084

[64] Hua, S. et al. High expression of GALNT7 promotes invasion and proliferation of glioma cells. *Oncol. Lett.* **16**, 6307–6314 (2018). 30405766 10.3892/ol.2018.9498 PMC6202485

[65] Lin, T. C. et al. GALNT6 expression enhances aggressive phenotypes of ovarian cancer cells by regulating EGFR activity. *Oncotarget* **8**, 42588 (2017). 28388560 10.18632/oncotarget.16585 PMC5522090

[66] Detarya, M. et al. The O-GalNAcylating enzyme GALNT5 mediates carcinogenesis and progression of cholangiocarcinoma via activation of AKT/ERK signaling. *Glycobiology* **30**, 312–324 (2020). 31868214 10.1093/glycob/cwz098

