# Supplementary material for: Targeting the proliferation of glioblastoma cells and enhancement of doxorubicin and temozolomide cytotoxicity through inhibition of PFKFB4 and HMOX1 genes with siRNAs
Source: Sci Rep. 2025 Jul 30;15:27861. doi: 10.1038/s41598-025-97192-z (PMC12311046; doi:10.1038/s41598-025-97192-z)
Supplement: Supplementary file 1 — Supplementary Material 1 [file 41598_2025_97192_MOESM1_ESM.docx]

Supplementary Tables

**Genes Included in Qiagen’s Human Cell Death Pathway PCR Array**

| **Table S1.** Human Cell Death Pathway PCR array genes (Qiagen, USA). | | |
| --- | --- | --- |
| **Symbol** | **Description** | **Unigene** |
| ABL1 | C-abl oncogene 1, non-receptor tyrosine kinase | Hs.431048 |
| AKT1 | V-akt murine thymoma viral oncogene homolog 1 | Hs.525622 |
| APAF1 | Apoptotic peptidase activating factor 1 | Hs.552567 |
| APP | Amyloid beta (A4) precursor protein | Hs.434980 |
| ATG12 | ATG12 autophagy related 12 homolog (S. cerevisiae) | Hs.264482 |
| ATG16L1 | ATG16 autophagy related 16-like 1 (S. cerevisiae) | Hs.529322 |
| ATG3 | ATG3 autophagy related 3 homolog (S. cerevisiae) | Hs.477126 |
| ATG5 | ATG5 autophagy related 5 homolog (S. cerevisiae) | Hs.486063 |
| ATG7 | ATG7 autophagy related 7 homolog (S. cerevisiae) | Hs.740389 |
| ATP6V1G2 | ATPase, H+ transporting, lysosomal 13kDa, V1 subunit G2 | Hs.249227 |
| BAX | BCL2-associated X protein | Hs.624291 |
| BCL2 | B-cell CLL/lymphoma 2 | Hs.150749 |
| **Table S1** Continue. | | |
| BCL2A1 | BCL2-related protein A1 | Hs.227817 |
| BCL2L1 | BCL2-like 1 | Hs.516966 |
| BCL2L11 | BCL2-like 11 (apoptosis facilitator) | Hs.469658 |
| BECN1 | Beclin 1, autophagy related | Hs.716464 |
| BIRC2 | Baculoviral IAP repeat containing 2 | Hs.696238 |
| BIRC3 | Baculoviral IAP repeat containing 3 | Hs.127799 |
| BMF | Bcl2 modifying factor | Hs.591104 |
| C1orf159 | Chromosome 1 open reading frame 159 | Hs.235095 |
| CASP1 | Caspase 1, apoptosis-related cysteine peptidase (interleukin 1, beta, convertase) | Hs.2490 |
| CASP2 | Caspase 2, apoptosis-related cysteine peptidase | Hs.368982 |
| CASP3 | Caspase 3, apoptosis-related cysteine peptidase | Hs.141125 |
| CASP6 | Caspase 6, apoptosis-related cysteine peptidase | Hs.654616 |
| CASP7 | Caspase 7, apoptosis-related cysteine peptidase | Hs.9216 |
| CASP9 | Caspase 9, apoptosis-related cysteine peptidase | Hs.329502 |
| CCDC103 | Coiled-coil domain containing 103 | Hs.743398 |
| CD40 | CD40 molecule, TNF receptor superfamily member 5 | Hs.472860 |
| CD40LG | CD40 ligand | Hs.592244 |
| CFLAR | CASP8 and FADD-like apoptosis regulator | Hs.390736 |
| **Table S1** Continue. | | |
| COMMD4 | COMM domain containing 4 | Hs.351327 |
| CTSB | Cathepsin B | Hs.520898 |
| CTSS | Cathepsin S | Hs.181301 |
| CYLD | Cylindromatosis (turban tumor syndrome) | Hs.578973 |
| DEFB1 | Defensin, beta 1 | Hs.32949 |
| DENND4A | DENN/MADD domain containing 4A | Hs.654567 |
| DFFA | DNA fragmentation factor, 45kDa, alpha polypeptide | Hs.484782 |
| DPYSL4 | Dihydropyrimidinase-like 4 | Hs.100058 |
| EIF5B | Eukaryotic translation initiation factor 5B | Hs.158688 |
| ESR1 | Estrogen receptor 1 | Hs.744830 |
| FAS | Fas (TNF receptor superfamily, member 6) | Hs.667309 |
| FASLG | Fas ligand (TNF superfamily, member 6) | Hs.2007 |
| FOXI1 | Forkhead box I1 | Hs.87236 |
| GAA | Glucosidase, alpha; acid | Hs.1437 |
| GADD45A | Growth arrest and DNA-damage-inducible, alpha | Hs.80409 |
| GALNT5 | UDP-N-acetyl-alpha-D-galactosamine:polypeptide N-acetylgalactosaminyltransferase 5  (GalNAc-T5) | Hs.269027 |
| GRB2 | Growth factor receptor-bound protein 2 | Hs.444356 |
| **Table S1** Continue. | | |
| HSPBAP1 | HSPB (heat shock 27kDa) associated protein 1 | Hs.29169 |
| HTT | Huntingtin | Hs.518450 |
| IFNG | Interferon, gamma | Hs.856 |
| IGF1 | Insulin-like growth factor 1 (somatomedin C) | Hs.160562 |
| IGF1R | Insulin-like growth factor 1 receptor | Hs.643120 |
| INS | Insulin | Hs.700350 |
| IRGM | Immunity-related GTPase family, M | Hs.519680 |
| JPH3 | Junctophilin 3 | Hs.592068 |
| KCNIP1 | Kv channel interacting protein 1 | Hs.484111 |
| MAG | Myelin associated glycoprotein | Hs.643440 |
| MAP1LC3A | Microtubule-associated protein 1 light chain 3 alpha | Hs.632273 |
| MAPK8 | Mitogen-activated protein kinase 8 | Hs.138211 |
| MCL1 | Myeloid cell leukemia sequence 1 (BCL2-related) | Hs.632486 |
| NFKB1 | Nuclear factor of kappa light polypeptide gene enhancer in B-cells 1 | Hs.618430 |
| NOL3 | Nucleolar protein 3 (apoptosis repressor with CARD domain) | Hs.513667 |
| OR10J3 | Olfactory receptor, family 10, subfamily J, member 3 | Hs.553833 |
| PARP1 | Poly (ADP-ribose) polymerase 1 | Hs.177766 |
| PARP2 | Poly (ADP-ribose) polymerase 2 | Hs.409412 |
| **Table S1** Continue. | | |
| PIK3C3 | Phosphoinositide-3-kinase, class 3 | Hs.656958 |
| PVR | Poliovirus receptor | Hs.171844 |
| RAB25 | RAB25, member RAS oncogene family | Hs.632469 |
| RPS6KB1 | Ribosomal protein S6 kinase, 70kDa, polypeptide 1 | Hs.463642 |
| S100A7A | S100 calcium binding protein A7A | Hs.442337 |
| SNCA | Synuclein, alpha (non A4 component of amyloid precursor) | Hs.21374 |
| SPATA2 | Spermatogenesis associated 2 | Hs.48513 |
| SQSTM1 | Sequestosome 1 | Hs.587290 |
| SYCP2 | Synaptonemal complex protein 2 | Hs.202676 |
| MACO1 | Macoilin 1 | Hs.189782 |
| TNF | Tumor necrosis factor | Hs.241570 |
| TNFRSF10A | Tumor necrosis factor receptor superfamily, member 10a | Hs.591834 |
| TNFRSF11B | Tumor necrosis factor receptor superfamily, member 11b | Hs.81791 |
| TNFRSF1A | Tumor necrosis factor receptor superfamily, member 1A | Hs.713833 |
| TP53 | Tumor protein p53 | Hs.437460 |
| TRAF2 | TNF receptor-associated factor 2 | Hs.522506 |
| TXNL4B | Thioredoxin-like 4B | Hs.134406 |
| ULK1 | Unc-51-like kinase 1 (C. elegans) | Hs.47061 |
| **Table S1** Continue. | | |
| XIAP | X-linked inhibitor of apoptosis | Hs.356076 |
| ACTB | Actin, beta | Hs.520640 |
| B2M | Beta-2-microglobulin | Hs.534255 |
| GAPDH | Glyceraldehyde-3-phosphate dehydrogenase | Hs.592355 |
| HPRT1 | Hypoxanthine phosphoribosyltransferase 1 | Hs.412707 |
| RPLP0 | Ribosomal protein, large, P0 | Hs.546285 |

**The Percentages of wound closure for different time point with different treatment**

**Table S2.** The Percent of wound closure after treatment with siPFKFB4, siPFKFB4/DOX, and siPFKFB4/TMZ on U87-MG cells compared with the NC group.

|  | **NC** | | | **siPFKFB4** | | | **siPFKFB4/DOX** | | | **siPFKFB4/TMZ** | | |
| --- | --- | --- | --- | --- | --- | --- | --- | --- | --- | --- | --- | --- |
|  | **mean** | **SEM** | **N** | **mean** | **SEM** | **N** | **mean** | **SEM** | **N** | **mean** | **SEM** | **N** |
| **24 h** | 68.24% | 1.33% | 3 | 38.38% | 1.50% | 3 | **23.44%** | **1.16%** | **3.00** | 45.97% | 1.63% | 3 |
| **48 h** | 88.24% | 1.33% | 3 | 54.04% | 0.96% | 3 | **27.44%** | **1.16%** | **3.00** | 54.64% | 0.99% | 3 |
| **72 h** | 98.61% | 0.52% | 3 | 66.71% | 1.04% | 3 | **34.59%** | **1.02%** | **3.00** | 73.30% | 0.28% | 3 |

**Table S3.** The Percent of wound closure after treatment with siHMOX1, siHMOX1/DOX, and siHMOX1/TMZ on U87-MG cells compared with the NC group.

|  | **NC** | | | **siHMOX1** | | | **siHMOX1/DOX** | | | **siHMOX1/TMZ** | | |
| --- | --- | --- | --- | --- | --- | --- | --- | --- | --- | --- | --- | --- |
|  | **mean** | **SEM** | **N** | **mean** | **SEM** | **N** | **mean** | **SEM** | **N** | **mean** | **SEM** | **N** |
| **24 h** | 68.24% | 1.33% | 3 | 28.54% | 1.17% | 3 | **16.67%** | **0.61%** | **3** | 29.01% | 0.96% | 3 |
| **48 h** | 88.24% | 1.33% | 3 | 34.68% | 0.73% | 3 | **20.01%** | **0.87%** | **3** | 43.19% | 0.89% | 3 |
| **72 h** | 98.61% | 0.52% | 3 | 55.54% | 0.58% | 3 | **25.01%** | **0.87%** | **3** | 65.94% | 0.72% | 3 |

**Table S4.** The Percent of wound closure after treatment with DOX, and TMZ on U87-MG cells compared with the NC group.

|  | **NC** | | | **DOX** | | | **TMZ** | | |
| --- | --- | --- | --- | --- | --- | --- | --- | --- | --- |
|  | **mean** | **SEM** | **N** | **mean** | **SEM** | **N** | **mean** | **SEM** | **N** |
| **24 h** | 68.24% | 1.33% | 3 | **43.96%** | **0.72%** | **3** | **57.41%** | **0.95%** | **3** |
| **48 h** | 88.24% | 1.33% | 3 | **57.96%** | **0.95%** | **3** | **73.74%** | **0.63%** | **3** |
| **72 h** | 98.61% | 0.52% | 3 | **75.41%** | **1.31%** | **3** | **82.96%** | **0.95%** | **3** |

**Table S5.** Up-regulated genes **(A)** and down-regulated genes **(B)** in U87-MG treated with siPFKFB4 100nM/DOX IC_50_.

| **A** | | | **B** | | |
| --- | --- | --- | --- | --- | --- |
| **Gene**  **Symbol** | **Fold**  **Regulation** | **GO** | **Gene**  **Symbol** | **Fold**  **Regulation** | **GO** |
| DPYSL4 | 2.23 | regulates apoptosis | BCL2 | -2.65 | anti-apoptotic |
| GALNT5 | 2.25 | signal transduction | PARP2 | -2.01 | repair of DNA |
| TNFRSF10A | 3.93 | regulates apoptosis |  |  |  |

GO: Gene Ontology

**Table S6.** Up-regulated genes **(A)** and down-regulated genes **(B)** in U87-MG treated with DOX IC_50_ alone.

| **A** | | | **B** | | |
| --- | --- | --- | --- | --- | --- |
| **Gene**  **Symbol** | **Fold**  **Regulation** | **GO** | **Gene**  **Symbol** | **Fold**  **Regulation** | **GO** |
| BCL2A1 | 8.20 | anti-apoptotic | DPYSL4 | -2.82 | regulates apoptosis |
| COMMD4 | 2.36 | repair of DNA |  |  |  |
| FAS | 4.39 | regulates apoptosis |  |  |  |
| TNF | 2.45 | regulates apoptosis |  |  | 5gv  55 |
| TNFRSF10A | 2.35 | regulates apoptosis |  |  |  |

GO: Gene Ontology

**Table S7.** Up-regulated genes **(A)** and down-regulated genes **(B)** in U87-MG treated with siHMOX1 100nM.

| **A** | | | **B** | | |
| --- | --- | --- | --- | --- | --- |
| **Gene**  **Symbol** | **Fold**  **Regulation** | **GO** | **Gene**  **Symbol** | **Fold**  **Regulation** | **GO** |
| BCL2 | 4.13 | anti-apoptotic | CCDC103 | -2.28 | stabilize microtubules |
| BCL2A1 | 9.91 | anti-apoptotic | DPYSL4 | -2.16 | regulates apoptosis |
| BIRC2 | 2.44 | regulates apoptosis |  |  |  |
| CASP3 | 2.67 | regulates apoptosis |  |  |  |
| COMMD4 | 2.97 | repair of DNA |  |  |  |
| CTSS | 2.02 | regulates apoptosis |  |  |  |
| CYLD | 2.16 | regulates cell cycle |  |  |  |
| FAS | 5.08 | regulates apoptosis |  |  |  |
| MAG | 3.99 | regulation of astrocyte |  |  |  |
| MAPK8 | 2.64 | signal transduction |  |  |  |
| PVR | 2.06 | cell adhesion |  |  |  |
| TNF | 2.50 | regulates apoptosis |  |  |  |
| TRAF2 | 2.16 | regulates apoptosis |  |  |  |
| XIAP | 2.12 | regulates cell cycle |  |  |  |

GO: Gene Ontology
