# Supplementary material for: Targeting the proliferation of glioblastoma cells and enhancement of doxorubicin and temozolomide cytotoxicity through inhibition of PFKFB4 and HMOX1 genes with siRNAs
Source: Sci Rep. 2025 Jul 30;15:27861. doi: 10.1038/s41598-025-97192-z (PMC12311046; doi:10.1038/s41598-025-97192-z)
Supplement: Supplementary file 2 — Supplementary Material 2 [file 41598_2025_97192_MOESM2_ESM.pdf]

KEGG Database Project
Kanehisa Laboratories
www.kanehisa.jp

Ref: 250094

Permission is granted to Scientific Reports of Springer Nature Ltd to publish both in print and digital under the CC BY 4.0 open access license the following KEGG pathway map image in the article "Targeting The Proliferation of Glioblastoma Cells and Enhancement of Doxorubicin and Temozolomide Cytotoxicity Through Inhibition of PFKFB4 and HMOX1 Genes with siRNAs" written by Hamzeh J. Al-Ameer and colleagues:

- Apoptosis - Homo sapiens (human) (hsa04210)

subject to the condition that the original source is acknowledged by citing at least one KEGG paper.

Permission granted: Naomi Takahashi

Naomi Takahashi, Kanehisa Laboratories

Date: 16 January 2025

Copyright holder: Kanehisa Laboratories
